# Pure Gaussian Apodisation Reduces Integral Crosstalk Sensitivity in Quantitative NMR

**DOI:** 10.1002/mrc.70113

**Published:** 2026-05-04

**Authors:** A. Flook, C. S. Raman, G. C. Lloyd‐Jones

**Affiliations:** ^1^ School of Chemistry University of Edinburgh Edinburgh UK; ^2^ Department of Pharmaceutical Sciences, School of Pharmacy University of Maryland Baltimore Maryland USA

## Abstract

Exponential apodisation is routinely applied in quantitative NMR measurements to increase signal‐to‐noise ratio (SNR) and integral precision, with a peak broadening effect. However, apodisation suitability is typically evaluated using SNR and peak broadening metrics and does not consider how apodisation alters peak area distribution. Here, we assess how exponential apodisation extends peak wings and relocates area away from the peak centre. This necessitates larger integral regions to account for the same area, increasing the potential for peak overlap and introducing variability in the trueness of the measurement. As an alternative, we show that a pure Gaussian apodisation can deliver comparable SNR improvements to the common exponential apodisation when tuned to a matched broadening criterion, reducing overlap of peak wings and improving robustness of finite‐window integration. As a result, the narrower line width prior to apodisation can be used to guide appropriate integral region choice. Overall, a pure Gaussian apodisation is demonstrated to be a robust alternative to exponential apodisation, especially in conjunction with complex 1D NMR spectra.

## Graphical abstract

Exponential apodisation redistributes peak area away from its centre, which increases crosstalk with neighbouring peaks and reduces integral trueness when integrating between defined regions. A pure Gaussian apodisation limits wing growth: Crosstalk between peaks is not worsened, and trueness is conserved during integration.

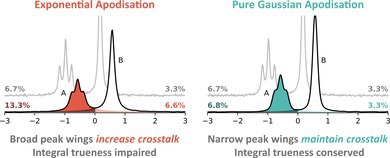

1

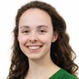

**Annabel Flook—MRC award at the SMASH Conference 2025**

Annabel completed her Masters in Chemistry under the supervision of Dr. John Lowe, Dr. Catherine Lyall and Dr. David Carbery at the University of Bath, where she was first introduced to NMR for reaction monitoring. Continuing on a similar theme, she began her PhD in 2022 with Guy Lloyd‐Jones FRS at the University of Edinburgh. Her research focuses on developing signal processing methods and data analysis workflows for reaction monitoring by NMR, with the aim of maximising the information that can be extracted from existing data and making these techniques accessible to the non‐NMR specialist.

## 2 Introduction

NMR for quantitative analysis (qNMR) has become a well‐established analytical technique across a broad disciplinary range including metabolomics [1, 2], fraud prevention [3, 4, 5], reaction monitoring [6, 7, 8] and isotopic analysis [9]. The breadth of application is testament to the ease at which quantitative measurements can be obtained without specific NMR expertise, especially as automated data acquisition and processing workflows have developed [10, 11, 12].

The intuitive, direct relationship between peak integrals in qNMR and number of nuclei in the sample provides access to quantitative concentration data without the need for calibration curves. Nevertheless, care must be taken when choosing both acquisition and processing parameters to ensure these measured integrals are quantitative, as different choices (zero‐filling, apodisation and integral limits) contribute quantifiable measurement uncertainty [13, 14, 15, 16, 17, 18, 19, 20, 21, 22]. Well‐informed acquisition parameter choices aid assessment of measurement trueness and precision and as such, there is significant literature describing the requirements for acquiring quantitative 1D spectra. Such guides also emphasise the importance of careful processing, including phase and baseline corrections, the requirement for either large integration regions (often > 20xFWHH) [23, 24] or lineshape modelling approaches to measure peak integrals [25, 26] and suitable signal‐to‐noise ratio (SNR) thresholds for precise measurements (SNR > 150 for 1% error) [14]. Within a typical processing workflow, apodisations (also referred to as window functions or windowing) are often used to improve measurement precision [23, 27, 28, 29, 30, 31].

Apodisations are mathematical functions that are typically multiplied with the time‐domain free induction decay (FID) prior to Fourier transform. The effect of the apodisation on the spectrum following Fourier transform varies depending on the chosen function [30, 32]. While not mutually exclusive, apodisations generally either (1) increase SNR by suppressing noise towards the end of the acquisition which improves integral precision, (2) enhance spectral resolution by artificially slowing the FID decay, narrowing linewidths and aiding resolution of splitting patterns and overlapped peaks, or (3) minimise artefacts from truncated FIDs, which would otherwise hinder analysis and integration by artificially forcing the FID to zero.

While a selection of functions is readily available to users of commercial software through checkboxes and parameter modification, exponential apodisation is advised for qNMR measurements and is often applied by commercial software as part of default 1D NMR spectral processing [21, 33].

**FIGURE 1:**
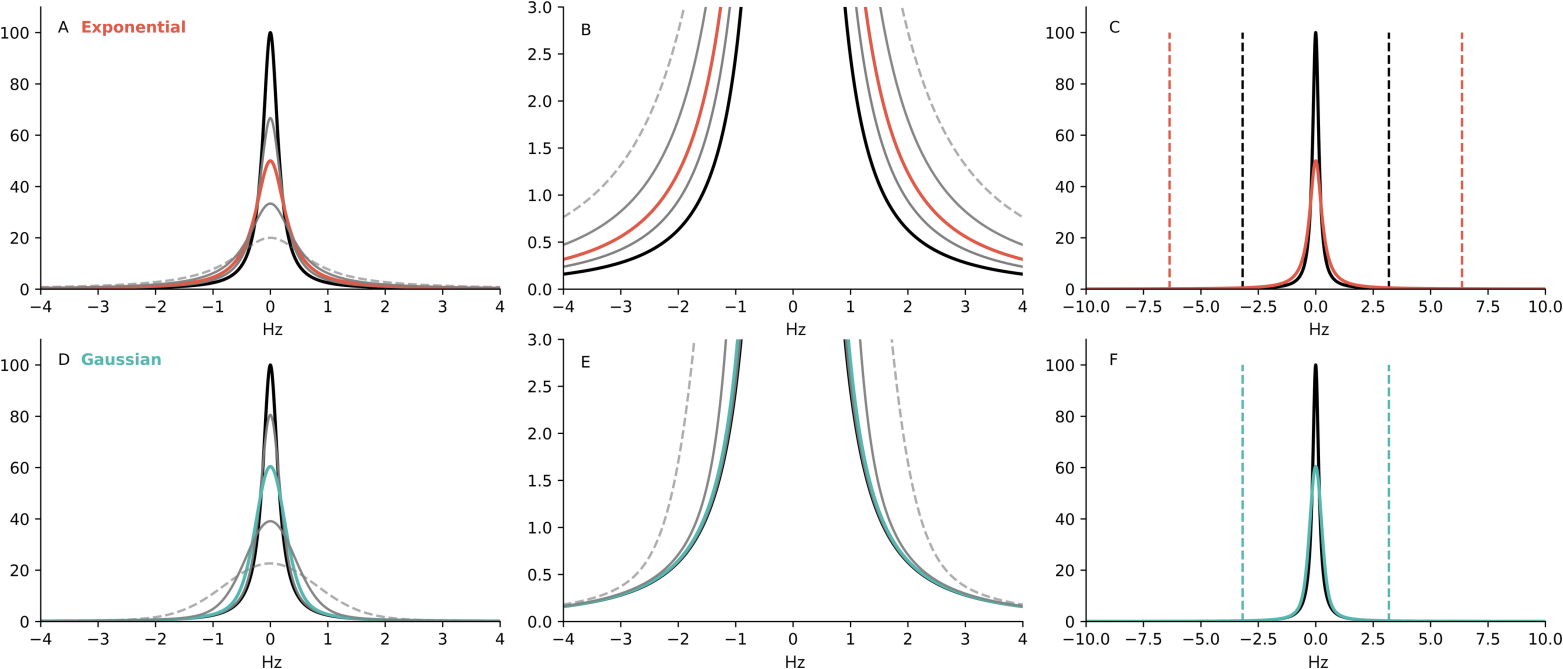
Singlet prior to (black, bold) and after apodisation (top: exponential, bottom: Gaussian). Natural FID decay: 1 Hz. Exponential apodisation (lb): 0.5 s^−1^, 1 s^−1^ (red), 2 s^−1^and 4 s^−1^ (dashed). Gaussian apodisation (gb, calculated with Equation (4)): 0.17 s^−2^, 0.69 s^−2^ (blue), 2.77 s^−2^, 11 s^−2^ (dashed). Lines in red and blue represent apodisation doubling FWHM. (A, B) Exponential apodisation cause peak wings to broaden and peak area to be dispersed away from the centre. (C) Integral regions for 96% of total integral prior to apodisation (black) and following exponential apodisation (1 Hz, red). (D, E) Gaussian apodisation induce FWHM broadening but peak wings are not broadened. (F) Integral regions for 96% of total integral prior to apodisation and following Gaussian apodisation (0.69 s^−2^) overlap (blue).

Exponential apodisation involves multiplication of the natural FID, which decays with rate $R_{2}^{*}$, with an exponential decay function with a ‘line broadening’ parameter, ‘*lb*’ (Equation (1)). The latter portion of the FID containing noise is suppressed and the noise in the spectrum is consequentially minimised while maintaining the characteristic Lorentzian peak shape seen in an idealised NMR spectrum (Figure 1A,B). An optimal SNR can be obtained when *lb* matches the natural decay of the FID, referred to as the ‘matched filter’ (Figure 1, red line).

(1) $$\mathrm{FI}D_{E}=e^{-\mathrm{i\omega t}}\cdot e^{-R_{2}^{*}t}\cdot e^{-\left(\mathrm{lb}\right)t}$$

Following exponential apodisation, the Lorentzian peak shape is broadened. This is described in terms of the full width at half maximum (FWHM) (Equation (2)): The FWHM is doubled when the matched filter is applied.

(2) $$\begin{array}{c} \text{FWHM}\;\left(\mathrm{Hz}\right)=\frac{R_{2}^{*}+\mathrm{lb}}{\pi } \end{array}$$

However, when integral measurements are of interest, the entire peak shape must be considered. Lorentzian functions belong to the Cauchy distribution family, meaning a significant portion of the area under the peak resides in wings which decay with 1/x^2^: a power law that extends to infinity. An exponential apodisation causes the integral to be redistributed away from the peak centre. When measuring peak areas by integration, the consequence is twofold. Firstly, the integral regions must be enlarged to account for the newly extended peak wings, as shown in Figure 1C (black and red lines) [34]. This is not always achievable in practical applications with spectral complexity and overlapped peaks. Secondly, the broadened peak wings have increased propensity to overlap with their neighbours, introducing an apodisation dependency to the integral measurements. The increased peak overlap from exponential apodisation distorts integrals in ways influenced by the proximity and prominence of neighbouring peaks and cannot necessarily be reliably or easily quantified. In summary, despite the benefits to precision offered by exponential apodisation, it is accompanied by a nonquantifiable and variable detriment to integral trueness.

Deconvolution methods can theoretically account for the overlap of Lorentzian peak wings, especially if a global spectral deconvolution (GSD) approach is taken. However, GSD introduces operator dependency, requires accurate initial parameter estimates, presents challenges when tackling complex baselines or nonideal lineshapes and is less accessible to a general user, especially when high‐performance models are desired [35].

More recently, a novel qNMR compliant apodisation has been proposed, but this relies on a more complex time‐domain filter designed primarily for resolution enhancement while preserving integrals [36]. The resolution‐enhancing Lorentz–Gauss apodisation has also been shown to be compatible with qNMR [34, 37] but does so without SNR optimisation and is controlled by two user‐defined parameters which must be carefully selected and assessed. While modest Lorentz–Gauss apodisation can be compatible with qNMR, aggressive resolution enhancement degrades trueness in practical application.

An alternative apodisation to enhance SNR takes a pure Gaussian form, where the apodisation decay is dependent only on a ‘Gaussian broadening’ parameter ‘*gb*’ and the square of time, *t* (Equation (3)) and is distinct from the Lorentz–Gauss transformation. Gaussian apodisations cause the Lorentzian peaks to take on a Gaussian component, producing Voigt peak shapes which are narrower at their base than their exponential counterparts (Figure 1D,E), and maintain peak area about their centres (Figure 1F).

Pure Gaussian apodisation functions are routinely available in NMR processing software. However, they are rarely used in qNMR for 1D spectral processing despite earlier work noting that Gaussian apodisation reduces bias when estimating peak areas and that it could be used in place of an exponential apodisation [27, 38, 39, 40]. However, these studies focus on idealised, isolated peaks and discuss FWHM and SNR, without addressing experimental realities such as finite integration regions and overlap with neighbouring peaks. As such, there is limited practical qNMR guidance that describes how SNR‐enhancing apodisation redistributes peak area outside finite integration regions and modulates overlap‐dependent integration bias in crowded spectra.

(3) $$\begin{array}{c} \mathrm{FI}D_{G}=e^{-\mathrm{i\omega t}}\cdot e^{-R_{2}^{*}t}\cdot e^{-\mathrm{gb}\cdot t^{2}} \end{array}$$

Here, we introduce a pure Gaussian apodisation broadening parameter, B, that improves SNR comparably to the standard exponential apodisation while minimising peak overlap. This maintains the trueness of measured integrals and allows integral regions to be defined by the narrower natural linewidth. A practical and quantitative guide can then be given on how to apply the pure Gaussian window as a conservative, integral friendly alternative to exponential in 1D qNMR of crowded spectra. Each aspect is demonstrated using peak area distribution alongside FWHM and SNR, and overall, we show that Gaussian apodisation is more suitable than the classical exponential function when integrating crowded spectra.

We focus our discussion below on integration‐based qNMR in spectra where peak overlap and finite integration regions dominate measurement error. Gaussian apodisation is applied as a linear time‐domain weighting. However, as with any apodisation method, the FID after apodisation must approach zero at the end of the acquisition time to avoid truncation artefacts that bias integral measurements. For workflows based on explicit lineshape fitting or digital reference standards, the applied Gaussian apodisation function should be included in the fitting because Lorentzian‐only models will be unsuitable for a Gaussian‐apodised spectrum [25].

## 3 Results and Discussion

### 3.1 SNR Enhancement of Gaussian and Exponential Apodisations

As exponential apodisations are employed to improve spectral SNR, it is necessary to identify a value for *gb* that increases SNR in an equivalent manner to the exponential apodisations for comparison and to provide a practical mathematical conversion between exponential and Gaussian settings.

**FIGURE 2:**
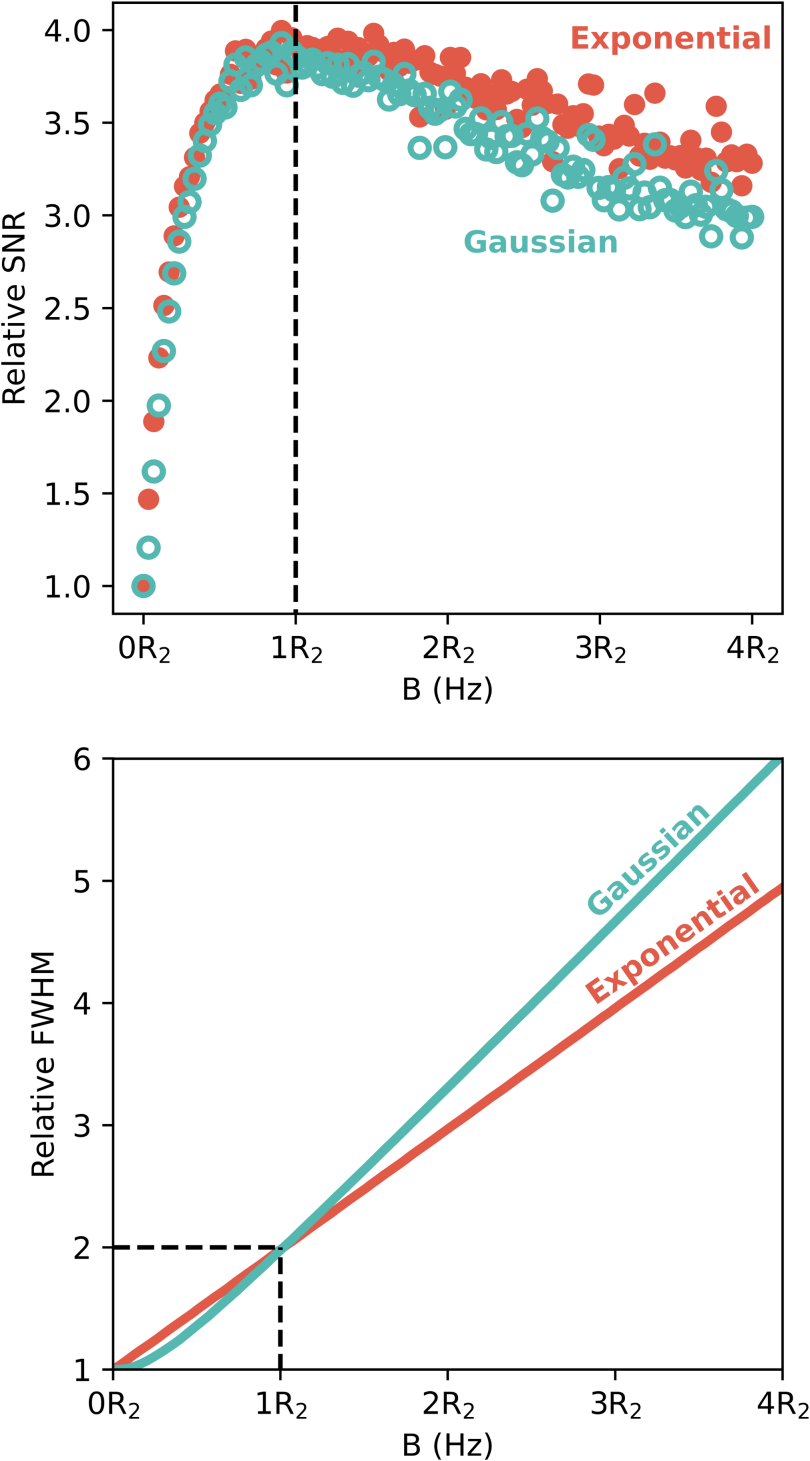
(top): Relative SNR and (bottom): Relative FWHM following Exponential (red) and Gaussian (blue) apodisation. Extent of apodisation described by broadening parameter, B, which is relative to FID decay ( $R_{2}^{*}$) and natural peak width (FWHM×π), and used in conjunction with Equations (4) and (5). Black dashed line represents a broadening parameter, B (Hz) = 1× $R_{2}^{*}$, which maximises SNR and doubles FWHM.

A simple relationship between the broadening parameter, B and *gb* can be identified (Equation (4)). A relationship exists between B and the FWHM of Voigt and Lorentzian peaks [41], and a convenient operating window can be found when both apodisations produce comparable FWHM broadening (see Supporting Information for details). Using this FWHM‐matching parameterisation, SNR exhibits an optimum when B is close to the natural FID decay, $R_{2}^{*}$, for both apodisations in the regimes tested. Here, the exponential broadening parameter B is the strict matched filter for Lorentzian lineshapes (Equation (5), lb. = $R_{2}^{*}$ = B) so that both apodisations double the natural FWHM. This case is depicted by the dashed lines in Figure 2.

(4) $$\mathrm{gb}=\ln\left(2\right)B^{2}$$

(5) $$\begin{array}{c} \mathrm{lb}=B \end{array}$$

The consequential doubling of FWHH can be disadvantageous to resolution, disguising splitting patterns and overlapping peaks. A compromise can be found with more conservative apodisation values: smaller values of *gb* provide equivalent SNR enhancement (Figure 2, top) as the exponential counterpart with a slightly narrower, although practically equivalent FWHM (Figure 2, bottom).

### 3.2 Exponential and Gaussian Peak Area Redistribution

**FIGURE 3:**
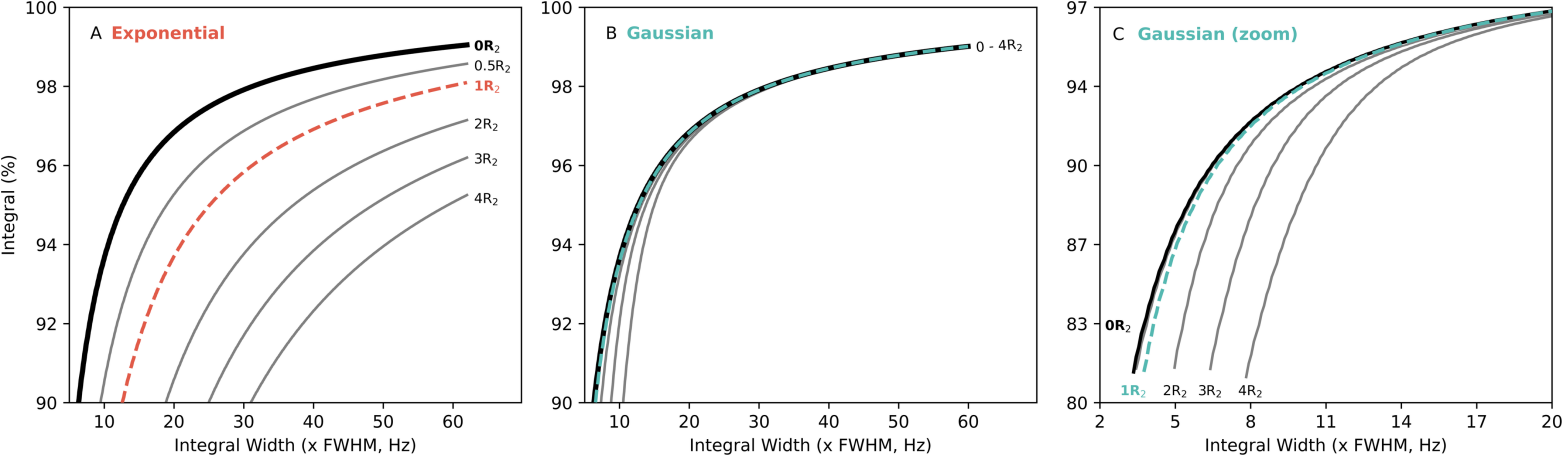
Integral (%) captured by integral regions defined as multiples of the FWHM prior to apodisation. Black line: no apodisation. (A) Following exponential apodisation, measured integral between predefined integral regions decreases. Red dashed line: exponential apodisation with matched filter (B (Hz) = 1× $R_{2}^{*}$). (B, C) Following Gaussian apodisation, measured integral is contained within predefined integral regions. Blue dashed line: Gaussian apodisation (B (Hz) = 1× $R_{2}^{*}$).

As shown in Figure 1C,F, the integral region must be redefined based on the extent of exponential apodisation (red and black dashed lines). However, this is not required when a Gaussian apodisation is employed: Appropriate integral regions can be defined prior to apodisation using the narrower natural FWHM and are not distorted by the Gaussian apodisation. Figure 3 depicts the proportion of integral measured by regions defined by the natural FWHM. Bolded black, red and blue lines depict the scenario shown in Figure 1.

With an exponential apodisation value chosen to maximise SNR (Figure 3A, red dashed line) and using 20 × FWHM, the peak area is distributed such that an additional 3% of the integral falls outside the predefined integral range. Conversely, the appropriate Gaussian apodisation produces 0.01% redistribution, a loss in trueness on the order of a hundred times less significant than when measurements with high trueness (~1%) are sought [13]. When spectral complexity necessitates smaller integral regions, a Gaussian apodisation to maximise SNR suffers only 0.10% integral redistribution with 10 × FWHM integral regions and 1.00% with 4.8 × FWHM.

Therefore, an integration‐accurate operating region can be defined for Gaussian apodisation when *B* ≤ 1, provided the apodised FID has decayed to a negligible proportion at the end of the acquisition time. Under these conditions, the proportion of area lost outside a 10 × FWHM window remains < 0.1%, and outside 20 × FWHM remains < 0.01%. For exponential apodisations, B must be negligible to achieve the same redistribution bound, and in this case, the SNR benefit is insignificant. Reference tables of values for both Gaussian and exponential apodisations are provided in the Supporting Information.

An approximation of $R_{2}^{*}$ is required to correctly calculate the apodisation parameters to maximise SNR, and $R_{2}^{*}$ can be estimated from the FWHM or FID decay. Smaller apodisation values naturally result in less area redistribution outside of the defined peak areas. However, it is interesting to demonstrate that apodisation values calculated after overestimation of $R_{2}^{*}$ result in only a marginal deviation in the expected integral measurement (Figure 3C). For example, even when B = 4 × R_2_, there is only a 0.22% area redistribution at 20 × FWHM.

### 3.3 Integral Crosstalk

While peak overlap is unavoidable in complex spectra, processing operations should aim to preserve trueness. However, exponential apodisation inherently causes area redistribution away from the peak centre and increases peak overlap. It is challenging to predict the increased extent of overlap, especially as multiple features of each peak exert an influence: proximity to its neighbours, shape defined by splitting patterns and coupling constants and relative areas. We introduce a crosstalk metric to quantify the overlap of one peak on its neighbour. For example, the crosstalk of peak A on the integral of peak B is defined as the integral of peak A found in integral region B as a percentage of peak B.

**FIGURE 4:**
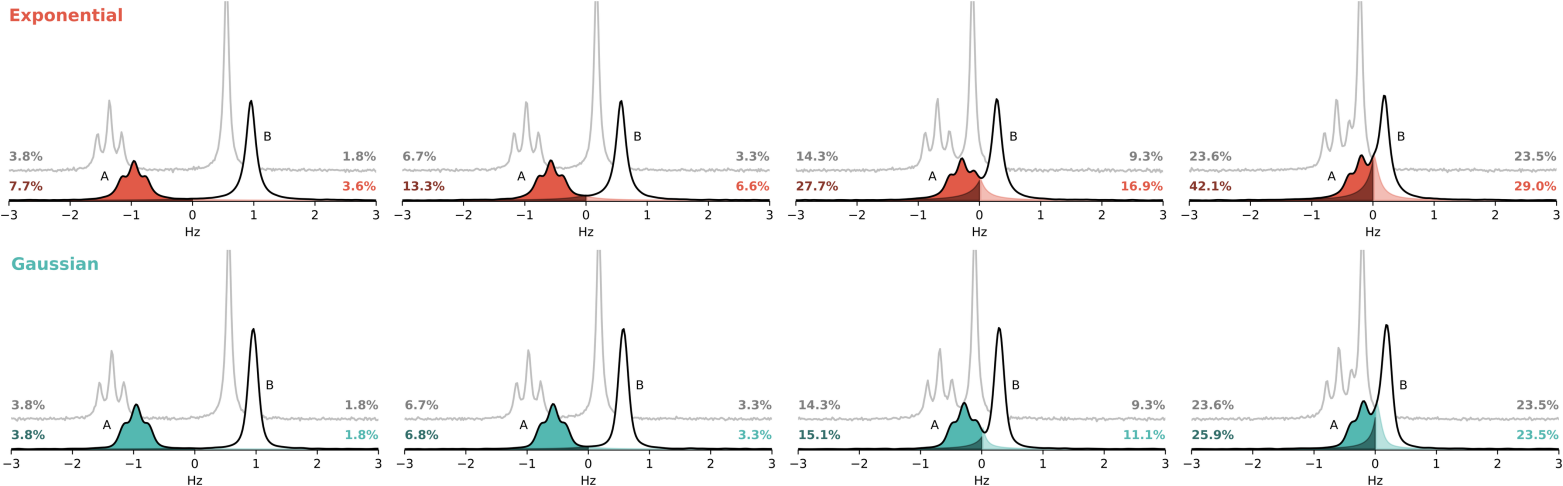
Simulated peaks (ratio: 2:3) prior to (grey) and after exponential apodisation (top, B= $R_{2}^{*}$) and Gaussian apodisation (bottom, B= $R_{2}^{*}$). Triplet coupling constant = 0.2 Hz, $R_{2}^{*}$ = 0.3 s ^− 1^, FWHM = $R_{2}^{*}$/π Hz, integral regions extend from 0 Hz to ‘10×FWHM + peak centre’ as peak overlap precludes usage of 20 × FWHM integral regions. Crosstalk of Peak A as a percent of Integral in Region B (shaded) prior to (grey) and after apodisation (bold). Distance between peak centres (Hz): 20 × FWHM, 12 × FWHM, 6 × FWHM, 4 × FWHM. Spectra offset to aid clarity of peak separation.

To provide an example, two peaks, with a ratio of 2:3, were simulated prior to (Figure 4, grey) and after apodisation to maximise SNR using B= $R_{2}^{*}$ (Figure 4, top). Prior to exponential apodisation, crosstalk is unavoidable, and it ranges from 2% (20 × FWHM separation) to 24% (4 × FWHM separation). Moreover, because the two peaks have different multiplicities, their areas are contained in different shapes and their crosstalk values are different. Following exponential apodisation (Figure 4, top), the crosstalk values increase as much as twofold and to differing extents. Therefore, exponential apodisation can induce ‘integral tuning’, in which smaller or larger default values of *lb* cause the measured integral to become unintentionally altered by a magnitude dependent on *lb*.

Conversely, the pure Gaussian apodisation does not significantly extend the peak area distribution (Figure 4, bottom) and has little influence over the crosstalk when peak separation is either 20 × FWHM or 12 × FWHM. As the distance between peaks is decreased to 6 × FWHM, the crosstalk induced from Gaussian apodisation is still minimal: compare 14.3% prior to and 15.1% following apodisation. The same conclusions can be understood by considering area redistribution following apodisation (Figure 3C) at integral regions of 4 × FWHM, which show minimal change of integral region prior to and after Gaussian apodisation.

In the most extreme case, 4 × FWHM separation as shown in the right‐hand side of Figure 4, the peaks A and B suffer substantially different changes in crosstalk following exponential apodisation. The Gaussian apodisation still maintains a similar crosstalk value, and the lack of change in singlet crosstalk is attributable to a slightly larger distribution of singlet area lying left of the 0 Hz centre than triplet redistribution towards the right of centre.

### 3.4 Apodisation in Crowded Spectra: Case Study 1

In reaction monitoring by NMR [6], the SNR increase brought by apodisation is valuable to identify and quantify low concentration species. However, the integral and proximity of each peak may change over the reaction lifetime. Exponential apodisation is expected to introduce small integration distortions in peaks of interest with dependency on the extension of peak wings from neighbouring signals. Conversely, the pure Gaussian function does not exacerbate the peak wings and is therefore expected to be more robust for one‐dimensional integration‐based qNMR. This will especially apply to complex or evolving mixtures where finite integration regions and partial overlap are intrinsic.

**FIGURE 5:**
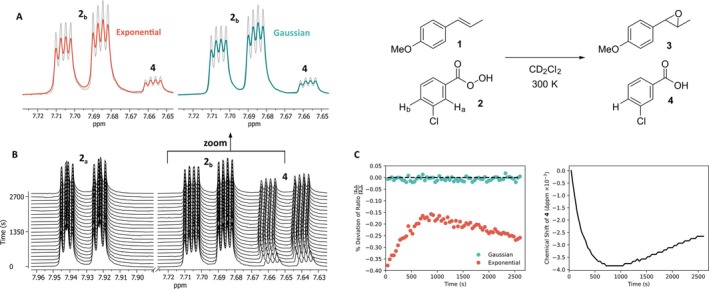
(A) The influence of exponential (left) and Gaussian (right) apodisation on the resolution of peaks **2**_**b**_ and **4** compared to prior apodisation (grey). (B) Stacked reaction monitoring spectra of the epoxidation of **1** (6 mM) with **2** (17 mM) after Gaussian apodisation for threefold SNR increase. (C, left): Difference in integral ratio (**2**_**b**_/**2**_**a**_) over the reaction lifetime following Gaussian (green) and exponential (red) apodisation. (C, right): Chemical shift change of m‐chlorobenzoic acid by‐product **4**. Gaussian and exponential apodisation parameters selected to enact a threefold SNR increase. No baseline correction was applied, and integral regions were identical in every case.

These differences in apodisation method became readily apparent in case study 1, where the epoxidation of *trans*‐anethole **1** with an excess of m‐chloroperoxybenzoic acid (mCPBA) **2** was monitored in situ by ^1^H NMR spectroscopy. In addition to the epoxide **3**, the process also generates acid **4** as a by‐product. The mCPBA presents two integrable peaks, one well resolved at 7.93 ppm (**2**_**a**_) and the other at 7.70 ppm (**2**_**b**_) in close proximity to a peak from acid **4**, whose integral and chemical shift change over the course of the reaction (Figure 5A,B). The SNR of the mCPBA was suitable to monitor the primary epoxidation process, but the SNR of the epoxide product **3** (SNR ≤ 3) was not sufficient to analyse its slow concurrent decomposition. In this case, the default exponential apodisation of 0.3 Hz resulted in a threefold SNR increase. It is not always feasible to maintain optimal shimming throughout monitoring due to the dynamic nature of the reaction medium, and the natural linewidths are already somewhat broadened and deviate from ideal Lorentzian peak shapes. The chosen SNR enhancement effect was deemed a conservative but adequate value to resolve low concentration peaks without excessive additional broadening.

To assess how apodisation could distort peak integrals, the deviation in the integral ratio **2**_**b**_ **/2**_**a**_ before and after apodisation over the reaction lifetime was explored (Figure 5C). Signal **2**_**a**_ was well resolved and was not expected to be influenced by crosstalk, but due to proximity to **4**, signal **2**_**b**_ was expected to show apodisation‐induced deviation that was dependent on the reaction if crosstalk was significant. The spectra were manually phased prior to integration and apodised in the absence of baseline correction to isolate the effects of apodisation from any deviation introduced by baseline correction procedures. The integral regions were chosen prior to apodisation and maintained for all analysis.

When exponential apodisation was applied, the ratio **2**_**b**_ **/2**_**a**_ initially saw 0.4% deviation away from the ratio prior to apodisation. The different peak shapes and intensities of **2**_**b**_ and **4** mean that crosstalk is not expected to be equal in both directions, and this result suggested that crosstalk of **2**_**b**_ into **4** was larger than the opposing crosstalk of **4** into **2**_**b**_. As the reaction progressed, the ratio **2**_**b**_ **/2**_**a**_ systematically appears to be minimised. However, these systematic changes can be attributed to the increased crosstalk of **4** into **2**_**b**_, reflected in the changing chemical shift of **4** (Figure 5C); initially, **2**_**b**_ is inflated and **2**_**b**_ **/2**_**a**_ appears truer (t < 1000 s) and then reduces (t > 1000 s). When baseline correction is applied in accordance to qNMR recommendations [42], the systematic deviation introduced by crosstalk is still observable (see Supporting Information).

When Gaussian apodisation was tuned to deliver a similar (approximately threefold) SNR enhancement, there was negligible deviation of the ratio **2**_**b**_ **/2**_**a**_ when compared to no apodisation (0.006%) and notably no evidence of systematic deviation caused by increased crosstalk with the neighbouring peak of **4** (Figure 5C).

### 3.5 Precision in Decongested Spectra: Case Study 2

Theoretical models suggest that the different peak profiles resulting from apodisation (Lorentzian, Gaussian and Voigt) have a significant effect on integral precision [43, 44]. Experimentally, exponential and Gaussian apodisations can both deliver high precision and accurate integrals in intentionally noncongested spectra, such as those designed for high precision kinetic isotope effects (KIE) measurements. For example, high precision is necessary to calculate heavy atom KIEs by ratiometric analysis of isotopologous substrates [13, 45]. The change in isotope ratio increases with conversion but becomes increasingly challenging to measure because of the corresponding decrease in the SNR of the substrates.

**FIGURE 6:**
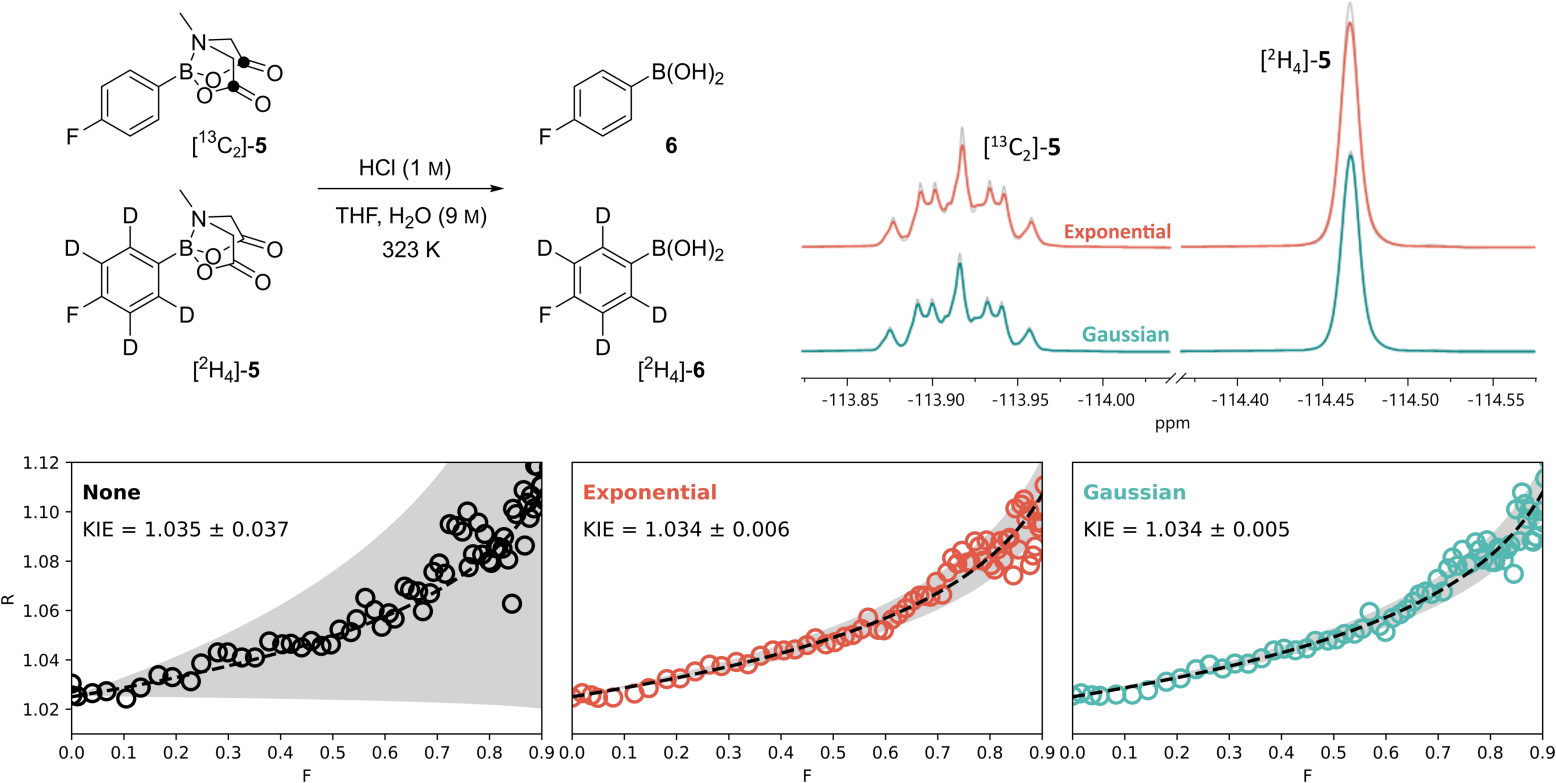
(A) Representative spectrum (t = 12 min) for the hydrolysis of [^*13*^C_2_]‐**5**/[^*2*^H_4_]‐**5** (R_0_ = 1.025) by HCl in a mixture of tetrahydrofuran (THF) and water monitored by in situ ^*19*^F NMR spectroscopy and 4‐scan averaging during postacquisition processing [46]. Exponential (red) and Gaussian (blue) apodisation were applied to enhance SNR by approximately threefold. Spectra are overlayed with the same spectrum without apodisation (grey) for reference. (B) Substrate ratio (R) plotted against fractional conversion (F) prior to apodisation (left) and following exponential (middle) and Gaussian (right) apodisation. Calculated KIE (black dashed line) derived from Bigeleisen–Wolfsberg [45] analysis of R/R_0_ between 0.2 ≤ F ≤ 0.9 (see Supporting Information S4.5 for equations). Shaded region defined by plotting KIE ±3σ.

The impact of exponential versus pure Gaussian apodisation on precision was explored in case study #2, where the ratiometric analysis of two methyliminodiacetic acetate (MIDA) boronate isotopologues was conducted. ^19^F NMR was chosen to remove potential overlap so that the precision of the two apodisation functions could be compared while trueness remained unaffected by crosstalk (Figure 6) [46, 47]. The SNR of the peaks of interest prior to apodisation (SNR of [^13^C_2_]‐**5** = 750 and SNR of [^2^H_4_]‐**5** = 330 at the beginning of the reaction) did not yield a ^13^C‐KIE with sufficient precision for mechanistic interpretation. Application of either apodisation function results in a threefold increase of SNR and yields a primary ^13^C/^12^C KIE with an acceptable error range (Figure 6).

Conversely to theoretical models [43, 44], this experimental example demonstrates that the altered peak profiles following Gaussian and exponential apodisation do not impact measurement precision under experimental conditions. Both apodisation functions produce similar precision and enhanced precision when compared to the data prior to apodisation. In these cases, pure Gaussian apodisation is not essential for successful quantification but remains a safe alternative to exponential apodisation.

### 3.6 Apodisation Effects in Congested Spectra: Case Study 3

NMR is widely applied in the analysis of very complex mixtures [1, 13]. These spectra are complicated by heavily overlapped regions, where peaks of interest span a large range of concentrations, and variable relaxation times produce both broad and narrow peaks. The complexity of these spectra renders integration between regions difficult and is generally avoided in favour of spectral deconvolution when quantifying these peaks.

**FIGURE 7:**
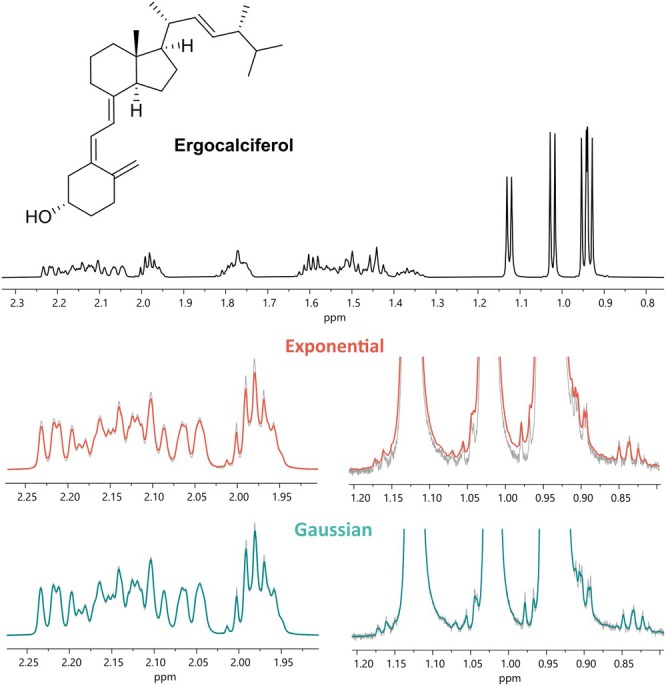
Partial ^1^H NMR spectrum of ergocalciferol and impurities, showing alkyl region between 0.8 and 2.3 ppm [49]. Exponential (red) and Gaussian (blue) apodisation were applied to enhance SNR by 3.8‐fold and overlaid with the spectrum prior to apodisation (grey).

The improved SNR from apodisation aids spectral fitting [11, 48], but as with less complex spectra, excessive apodisation broadens peaks without further gain in SNR. An apodisation value less than the FWHM of the narrowest peak suppresses noise at the end of the FID, maintaining resolution in highly overlapped regions and limiting unnecessary broadening. In the ^1^H NMR spectrum of ergocalciferol [49], exponential and Gaussian apodisation improved SNR by a factor of 3.8 and broadened the natural FWHM by only 33%. For both apodisation functions, the multiplet structure between 1.9 and 2.3 ppm is maintained (Figure 7, left).

The identification and quantification of minor peaks can be equally important as that of the major components, for example, in purity analysis [50]. Low intensity peaks can be disguised by larger neighbouring peaks and exacerbated by broadening. For example, minor peaks at 0.966 ppm are observable prior to apodisation but are almost consumed by the major ergocalciferol peaks following exponential apodisation (Figure 7, right). Additionally, the broadened wings of the major peaks dictate the shape of the surrounding baseline and may be erroneously removed during baseline correction. Broadening caused by the pure Gaussian apodisation is localised around the peak centre so that low intensity signals maintain resolution from the larger signals and the baseline is truer to the spectrum prior to apodisation.

This example demonstrates that a pure Gaussian apodisation can maintain resolution when large differences in intensities are present, unlike its exponential counterpart. Further qualitative assessment of the spectrum and the effects of apodisation may be necessary to reveal whether resolution has been compromised, especially when apodisation is applied in automatic processing regimes.

## 4 Conclusion

Exponential apodisation is frequently advised in qNMR, but the common justification of maximising SNR does not describe the consequences to peak area distribution, and increasing SNR does not dictate that trueness is maintained: an essential consideration alongside precision. We have extended existing analysis of the suitability of SNR‐enhancing apodisations by (1) quantifying the fraction of peak area lost outside fixed integration limits defined in units of the natural FWHM and (2) defining an ’integral crosstalk’ metric between neighbouring peaks as a function of separation and apodisation choice. These metrics directly expose how exponential apodisation can introduce apodisation‐dependent bias to integral measurements in crowded spectra, dependent on the line broadening value and spectral features such as proximity to neighbouring peaks.

A pure Gaussian apodisation offers a straightforward alternative by minimising peak area redistribution for a given SNR gain. Practically, the Gaussian apodisation allows narrower integral regions to be defined based on the FWHM of the spectrum prior to apodisation, with comparable SNR increase and line broadening penalty to the exponential function. In experimental scenarios, Gaussian apodisation rescues trueness in crowded spectra while maintaining the rigorous precision required for isotopic studies.

These results reposition apodisation from a discretionary postprocessing choice to a quantitatively critical design parameter in finite‐window qNMR integration. Adopting pure Gaussian apodisation has the potential to strengthen qNMR standards by providing pharmacopeial committees and metrology institutes with a mathematically explicit protocol that demonstrably suppresses the ‘integral tuning’ sensitivity introduced by exponential line broadening [49, 51, 52, 53]. This reduced crosstalk sensitivity shows that Gaussian apodisation can compress the effective spectral footprint of resonances and potentially reduce overlap‐driven integration errors in automated workflows when Lorentzian tails contribute substantially to measurement error [54]. Beyond solution‐state qNMR, the same overlap considerations arise in in vivo magnetic resonance spectroscopy, where metabolite quantitation from heavily overlapped resonances faces Lorentzian tail crosstalk challenges [55]. The strategy developed here may prove similarly beneficial in that domain, particularly when combined with explicit Voigt‐profile fitting.

## Author Contributions

**A. Flook:** conceptualization, investigation, writing – original draft, writing – review and editing, formal analysis. **C. S. Raman:** writing – review and editing, formal analysis. **G. C. Lloyd‐Jones:** writing – rspditing.

## Supporting information




**Table S1:** Reference table for peak area redistribution with exponential apodisation defined integral regions (× FWHM) and for broadening values (B). Integral region defined in terms of FWHM of non‐apodised peak. Some values of interested are highlighted.
**Table S2:** Reference table for peak area redistribution with pure Gaussian apodisation at defined integral regions (× FWHM) and for broadening values (B). Integral region defined in terms of FWHM of non‐apodised peak. Some values of interested are highlighted.
**Table S3:** Integration parameters used for raw data and averaged data.
**Table S4:** Integration parameters used for raw data and averaged data.
**Figure S1:** Simulated peaks (ratio: 2:3) prior to (grey) and after exponential apodisation (top, B = R_2_) and Gaussian apodisation (bottom, B = R_2_). Triplet coupling constant = 0.2 Hz, R_2_ = 0.3 s^−1^, FWHM = R_2_/π, integral regions extend from 0 Hz to ‘10×FWHM + peak centre’ as peak overlap precludes usage of 20 × FWHM integral regions. Crosstalk of Peak A as defined by alternative definition in Section S1.5. Distance between peak centres: 20 × FWHM, 12 × FWHM, 6 × FWHM, 4 × FWHM. Spectra offset to aid clarity of peak separation.
**Figure S2:** Idealised two‐peak geometry showing two Lorentzian resonances and the finite integration window $R_{B}$.
**Figure S3:** Closed‐form Lorentzian tail overlap $F_{A\to B}$ (fraction of resonance A falling within the finite integration window centred on resonance B) plotted versus exponential broadening strength $\frac{\mathrm{lb}}{R_{2}}$. The matched‐filter condition $\mathrm{lb}=R_{2}$ corresponds to $\frac{\mathrm{lb}}{R_{2}}=1$ (vertical dashed line), and the secondary axis indicates the associated linewidth scaling $\frac{\gamma_{E}}{\gamma_{0}}=1+\frac{\mathrm{lb}}{R_{2}}$.
**Figure S4:** Natural Lorentzian, exponential‐broadened Lorentzian, and Gaussian‐apodised Voigt under the same finite integration window (width in units of the natural FWHM), showing wing redistribution. See Table S1 and S2 for integral redistribution.
**Figure S5:** Absorptive‐real spectra for a single peak under: natural exponential (no extra window), exponential apodisation (target), Gaussian apodisation with c=ln2, and Gaussian apodization with $c=c_{\mathrm{opt}}$.
**Figure S6:** Stage A (left): $c_{\mathrm{opt}}$ vs. dt. Stage B (right): $c_{\mathrm{opt}}$ vs. $T_{\mathrm{acq}}$. Both estimators (‘linear’, ‘quadlog’) are shown.
**Figure S7:** FWHM mismatch (%) when using c=ln2: Stage A (left) vs. dt, Stage B (right) vs. $T_{\mathrm{acq}}$.
**Figure S8:** mrc70113‐sup‐0001‐Supporting_Information.docx. $c_{\mathrm{opt}}$ vs. $B/R_{2}$, with c=ln2 shown for reference.
**Figure S9:** FWHM mismatch (%) when using c=ln2 instead of $c_{\mathrm{opt}}$, plotted vs. $B/R_{2}$.
**Figure S10:** ENBW proxy vs. $B/R_{2}$ for exponential and Gaussian windows (using c=ln2 and $c=c_{\mathrm{opt}}$).
**Figure S11:** mrc70113‐sup‐0001‐Supporting_Information.docx. $Q_{\mathrm{rms}}$ vs. $B/R_{2}$ for exponential and Gaussian windows (using c=ln2 and $c=c_{\mathrm{opt}}$).
**Figure S12:** Estimator agreement vs. zero‐fill factor (log2): | $c_{\mathrm{opt}}$_linear − $c_{\mathrm{opt}}$_quadlog|, showing negligible dependence of inferred $c_{\mathrm{opt}}$ on FWHM estimator choice across zero‐filling (spectrum_mode = absorptivereal).
**Figure S13:** Concentration**‐**time profile of **2** and **4** over the reaction monitoring experiment.
**Figure S14:** Spectrum of Ergocalciferol. Natural FWHM estimated as 1.86 Hz using singlet at 0.634 ppm (indicated with arrow).

## Data Availability

The primary NMR data and scripts for data analysis associated with this work are available free of charge online (https://doi.org/10.7488/ds/8078).
