# Supplementary material for: Pure Gaussian Apodisation Reduces Integral Crosstalk Sensitivity in Quantitative NMR
Source: Magn Reson Chem. 2026 May 4;64(9):748–57. doi: 10.1002/mrc.70113 (PMC13432380; doi:10.1002/mrc.70113)
Supplement: Supplementary file 1 — Table S1: Reference table for peak area redistribution with exponential apodisation defined integral regions (× FWHM) and for broadening values (B). Integral region defined in terms of FWHM of non‐apodised peak. Some values of interested are highlighted. Table S2: Reference table for peak area redistribution with pure Gaussian apodisation at defined integral regions (× FWHM) and for broadening values (B). Integral region defined in terms of FWHM of non‐apodised peak. Some values of interested are highlighted. Table S3: Integration parameters used for raw data and averaged data. Table S4: Integration parameters used for raw data and averaged data. Figure S1: Simulated peaks (ratio: 2:3) prior to (grey) and after exponential apodisation (top, B = R2) and Gaussian apodisation (bottom, B = R2). Triplet coupling constant = 0.2 Hz, R2 = 0.3 s−1, FWHM = R2/π, integral regions extend from 0 Hz to ‘10×FWHM + peak centre’ as peak overlap precludes usage of 20 × FWHM integral regions. Crosstalk of Peak A as defined by alternative definition in Section S1.5. Distance between peak centres: 20 × FWHM, 12 × FWHM, 6 × FWHM, 4 × FWHM. Spectra offset to aid clarity of peak separation. Figure S2: Idealised two‐peak geometry showing two Lorentzian resonances and the finite integration window RB. Figure S3: Closed‐form Lorentzian tail overlap FA→B (fraction of resonance A falling within the finite integration window centred on resonance B) plotted versus exponential broadening strength lbR2. The matched‐filter condition lb=R2 corresponds to lbR2=1 (vertical dashed line), and the secondary axis indicates the associated linewidth scaling γEγ0=1+lbR2. Figure S4: Natural Lorentzian, exponential‐broadened Lorentzian, and Gaussian‐apodised Voigt under the same finite integration window (width in units of the natural FWHM), showing wing redistribution. See Table S1 and S2 for integral redistribution. Figure S5: Absorptive‐real spectra for a single peak under: natural exponential (no extra wind [file MRC-64-748-s001.docx]

**Supporting Information**

***Pure Gaussian Apodisation Reduces Integral Crosstalk Sensitivity in Quantitative NMR***

**A. Flook*, C.S. Raman, G. C. Lloyd-Jones**

**Table of Contents**

[S1. Assessment of the Gaussian broadening parameter 1](#_Toc226183072)

[S1.1 Definitions and assumptions 1](#_Toc226183073)

[S1.2. Derivation of Equation 4 (main text) and definition of B. 1](#_Toc226183074)

[S1.3. Practical safety check: avoid truncation ringing 4](#_Toc226183075)

[S1.4. Baseline correction interaction 5](#_Toc226183076)

[S1.5. Integral Crosstalk Definition 5](#_Toc226183077)

[S1.6. Closed-form overlap in an idealised two-peak model 6](#_Toc226183078)

[S1.7. Numerical audit of the mapping *gb = cB^2^* 8](#_Toc226183079)

[S1.8. Stage A: sampling and numerical convergence (varying dt) 10](#_Toc226183080)

[S1.9. Stage B: truncation and record length effects (varying T_acq_) 11](#_Toc226183081)

[S1.10. Parameter-regime sweep 11](#_Toc226183082)

[S1.12. Practical guidance 14](#_Toc226183083)

[S1.13. Practical implementations note: software parameterisation differs 14](#_Toc226183084)

[S2. Peak Area Redistribution Table 16](#_Toc226183085)

[S3. Case Study #1: Apodisation in Crowded Spectra 17](#_Toc226183086)

[S3.1 Stock Solutions 17](#_Toc226183087)

[S3.2 Spectrometer Details 17](#_Toc226183088)

[S3.3 Reaction Monitoring 17](#_Toc226183089)

[S3.4 NMR Processing Parameters 17](#_Toc226183090)

[S3.5 Data Analysis 18](#_Toc226183091)

[S4. Case Study #2: Maintaining Precision in Decongested Spectra 19](#_Toc226183092)

[S4.1 Stock Solutions 19](#_Toc226183093)

[S4.2 Spectrometer Details 19](#_Toc226183094)

[S4.3 Reaction Monitoring 19](#_Toc226183095)

[S4.4 NMR Processing Parameters 19](#_Toc226183096)

[S4.5 Data Analysis 20](#_Toc226183097)

[S5. Case Study #3: Apodisation Effects in Congested Spectra 21](#_Toc226183098)

[S6. Data Availability 22](#_Toc226183099)

[S7. References 22](#_Toc226183100)

# Assessment of the Gaussian broadening parameter

Whilst derivations of highly accurate Voigt-width approximations exist, ranging from classic empirical fits to modern ‘super‑accuracy’ schemes,^S1,S2^ this note aims to provide an operational protocol that reduces overlap-driven integration error while retaining near-optimal SNR.^S3,S4^ However, a pure Gaussian apodisation is not SNR-optimal in the strict matched-filter sense. It is advocated here as a conservative mismatch that can improve integration robustness (reduce overlap sensitivity) at a typically modest SNR penalty.

## S1.1 Definitions and assumptions

### Minimal FID model

For an isolated resonance, a standard idealised complex FID model for $t\geq0$ is defined in Eq. S1, where $R_{2}$ is the decay rate, $\omega_{0}$ is the resonance angular frequency, and $A$ is a complex amplitude.^S5,S6^ This model is sufficient to demonstrate the effects of apodisation on area redistribution but we acknowledge that it does not capture every experimental imperfection (eg imperfect shimming, baseline distortions, etc.).

|  | $s(t)=A e^{-i\omega_{0}t}e^{-R_{2}t}$ | Equation S1 |
| --- | --- | --- |

Apodisation is applied by multiplication to the FID in all cases. A mathematically equivalent approach is the convolution of the apodisation function with the spectrum following Fourier transform.

## S1.2. Derivation of Equation 4 (main text) and definition of B.

In the main text, we employ a broadening parameter B to identify values of gb and lb with comparable SNR and full width at half maximum (FWHM) of a peak (Eq. 4 and 5, main text). When $B=R_{2}$, FWHM increases 2-fold. However, the exact FWHM of a Voigt profile cannot be described by a simple relationship between the FWHM of the corresponding Gaussian and exponential components. Therefore, Equation 4 is a numerically identified approximation. In a practical setting, Equation 4 could be written as $gb=0.7B^{2}$.

However, approximations of the relationship between the FWHM of Gaussian and exponential components do exist. We use this relationship to demonstrate that Equation 4 is not arbitrary, and although more precise definitions exist, they are at a level that is not of additional practical use.

In the time domain (prior to Fourier transform), a Lorentzian profile is described by an exponential decay with decay rate constant, $k_{l}$. Note that, if $l\left( t \right)$ represents an FID, $A_{0}$ contains frequency information as well as initial amplitude. Its corresponding FWHM in the frequency domain has a linear relationship to $k_{l}$.

$$l\left( t \right)=A_{0}\exp\left( -k_{l}\cdot t \right); L_{\mathrm{FWHM}}=\frac{k_{l}}{\pi}$$

$L_{\mathrm{FWHM}}^{\mathrm{apod}}$ is obtained by increasing $L_{\mathrm{FWHM}}$ by exponential apodisation.

$$l^{\mathrm{apod}}\left( t \right)=l(t) exp \left( -k_{\mathrm{apod}}\cdot t \right); L_{\mathrm{FWHM}}^{\mathrm{apod}}=\frac{k_{l}+k_{\mathrm{apod}}}{\pi}$$

And $L_{\mathrm{FWHM}}^{\mathrm{apod}}={2L}_{\mathrm{FWHM}}$ when $k_{l}= k_{\mathrm{apod}}$

A pure Gaussian function in the time domain and prior to Fourier transform is described as the exponential decay with rate constant $k_{g}$ and the square of time, t. $k_{g}$ is also related to the FWHM in the frequency domain $G_{\mathrm{FWHM}}.$

$$g\left( t \right)=A_{0}\exp\left( -k_{g}\cdot t^{2} \right); G_{\mathrm{FWHM}}=\frac{2\sqrt{k_{g}}\sqrt{\ln\left( 2 \right)}}{\pi}$$

The multiplication of an exponential decay $l(t)$ with a pure Gaussian function $g(t)$ produces a Voigt function $v(t)$.

$$v\left( t \right)=l\left( t \right)\cdot g(t)$$

A simplistic approximation of the FWHM of a Voigt profile $V_{FWHM}$ is as a combination of $G_{\mathrm{FWHM}}$ and $L_{\mathrm{FWHM}}$ components.^S1^

$$V_{\mathrm{FWHM}}\cong\frac{L_{\mathrm{FWHM}}}{2}+\sqrt{\frac{L_{\mathrm{FWHM}}^{2}}{4}+G_{\mathrm{FWHM}}^{2}}$$

When $V_{\mathrm{FWHM}}=2\cdot L_{\mathrm{FWHM}}$, we can identify a relationship between $L_{\mathrm{FWHM}}$ and $G_{\mathrm{FWHM}}$:

$$2\left( L_{\mathrm{FWHM}} \right)^{2}\cong\left( G_{\mathrm{FWHM}} \right)^{2}$$

When rewritten in terms of $k_{l}$ and $k_{g}$, a relationship between the two rate constants can be identified.

$$\frac{4\left( k_{l} \right)^{2}}{\pi^{2}}\cong\frac{4{\ln\left( 2 \right)k}_{g}}{\pi^{2}}$$

$$\therefore\frac{\left( k_{l} \right)^{2}}{2\ln\left( 2 \right)}\cong k_{g}$$

$${{0.7(k}_{l})}^{2}\approx k_{g}$$

It must be reiterated here that $V_{FWHM}$ is an approximation with an associated error of approximately ± 10% associated error, but the squared relationship between $k_{l}$ and $k_{g}$ remains for better approximations.^S1^

We find numerically that $\ln\left( 2 \right)$ is a marginally better approximation and employ this in Equation 4, but generally the central claims are qualitative and operational (finite-window overlap sensitivity) and not contingent on a perfect mapping $gb=cB^{2}$.

In apodisation, $k_{l}$ and $k_{g}$ are chosen by the user and are referred to as the exponential apodisation broadening parameter $lb$ and the Gaussian apodisation broadening factor $gb$ respectively.

|  | $\begin{matrix} gb=\left( B \right)^{2}\ln\left( 2 \right) \end{matrix}$ | Equation 4 (main text) |
| --- | --- | --- |
|  | $\begin{matrix} \begin{matrix} lb=B \end{matrix} \end{matrix}$ | Equation 5 (main text) |

## S1.3. Practical safety check: avoid truncation ringing

If the FID ends while the window is still appreciably nonzero, the effective multiplication by a hard step at $T_{\mathrm{acq}}$ can introduce sinc-like ringing that can dominate integration errors.^S7^ This is not specific to Gaussian windows. Regardless, a simple, implementable safety condition is to require the Gaussian window to be below a small threshold $\varepsilon$ at the final acquired time:

|  | $w_{G}(T_{\mathrm{acq}})=exp(-gb T_{\mathrm{acq}}^{2})\leq\varepsilon$ | Equation S3 |
| --- | --- | --- |

With Equation 4 ($gb=B^{2}\ln2$), this becomes:

|  | $\exp\left( -ln2 (BT_{\mathrm{acq}})^{2} \right)\leq\varepsilon\Leftrightarrow BT_{\mathrm{acq}}\geq\sqrt{\frac{ln(1/\varepsilon)}{\ln2}}.$ | Equation S4 |
| --- | --- | --- |

**Rule of thumb (Gaussian).** If a threshold of $w_{G}(T_{\mathrm{acq}})\leq{10}^{-3}$ is required, enforce $BT_{\mathrm{acq}}\gtrsim3.16$; for $w_{G}(T_{\mathrm{acq}})\leq{10}^{-4}$, enforce $BT_{\mathrm{acq}}\gtrsim3.85$. If this criterion is not met, either reduce $B$ (weaker apodisation), increase $T_{\mathrm{acq}}$, or use a different processing strategy.

**Comparison to Exponential Apodisation**

With equivalent values of B, Gaussian functions tend to 0 faster than exponential functions. This means that, for a given truncated $T_{acq}$, a larger exponential apodisation is required to obtain the desired threshold than the equivalent Gaussian apodisation.

For comparison to an exponential apodisation:

|  | $w_{E}(T_{\mathrm{acq}})=exp(-lb T_{\mathrm{acq}})\leq\varepsilon$ | Equation S5 |
| --- | --- | --- |

With the matched filter ($lb=B$), this becomes:

|  | $\exp\left( -BT_{\mathrm{acq}} \right)\leq\varepsilon\Leftrightarrow BT_{\mathrm{acq}}\geq\ln\left( \frac{1}{\varepsilon} \right).$ | Equation S6 |
| --- | --- | --- |

**Rule of thumb (Exponential).** If a threshold of $w_{E}(T_{\mathrm{acq}})\leq{10}^{-3}$ is required, enforce $BT_{\mathrm{acq}}\gtrsim6.91$; for $w_{G}(T_{\mathrm{acq}})\leq{10}^{-4}$, enforce $BT_{\mathrm{acq}}\gtrsim9.21$.

## S1.4. Baseline correction interaction

The analytic “integral crosstalk” model (idealised two-peak case) assumes an ideal baseline over the integration region. In real workflows, baseline correction may partially absorb broad wings, and the extent of this absorption depends on the baseline method and its settings; therefore, measured crosstalk can be workflow dependent. Practical advantage can be validated under the baseline correction strategy used for the desired qNMR pipeline.

## S1.5. Integral Crosstalk Definition

Related effects have long been discussed in the NMR processing and quantitation literature under terms such as spectral overlap and truncation artefacts/bias; here we use the term “integral crosstalk” to give an explicit, finite-window contamination metric directly aligned with routine qNMR integration practice.

**Box 1 — Definition (integral crosstalk).**
For resonances A and B with spectra $S_{A}(\nu)$, $S_{B}(\nu)$, where $S_{total}\left( \nu\right)=S_{A}\left( \nu\right)+S_{B}\left( \nu\right)$ and integration region $\mathcal{R}_{B}$ intended for resonance B, define:

$$\mathrm{XT}_{A\to B}(\mathcal{R}_{B})=100\%\times\frac{\int_{\mathcal{R}_{B}} S_{A}(\nu) d\nu}{\int_{\mathcal{R}_{B}} S_{total}(\nu) d\nu}.$$

This definition reflects finite-window integration practice and quantifies neighbour contamination in percent units directly relevant to qNMR.

An alternative definition replaces $S_{total}\left( \nu\right)$ with $S_{B}\left( \nu\right)$, which rescales the equation presented in Box 1 and described how big the contaminant is compared with the analyte signal between the same integral region. For completeness, Figure 4 (main, text) can be recalculated using the alternative definition and is shown as Figure S1.


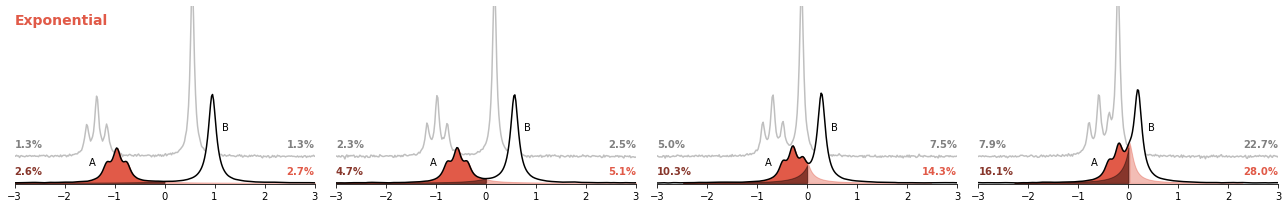

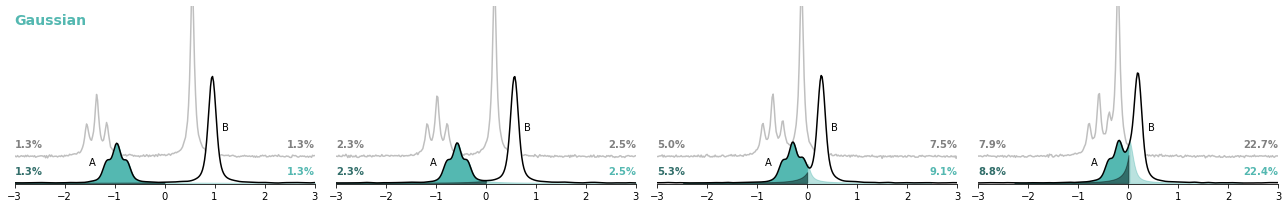


**Figure S1:** *Simulated peaks (Ratio: 2:3) prior to (grey) and after exponential apodisation (top, B=R_2_) and Gaussian apodisation (bottom, B=R_2_). Triplet coupling constant = 0.2 Hz, R_2_ = 0.3 s^-1^, FWHM = R_2_/π, integral regions extend from 0 Hz to “10×FWHM + peak centre” as peak overlap precludes usage of 20×FWHM integral regions. Crosstalk of Peak A as defined by alternative definition in Section S1.5. Distance between peak centres: 20×FWHM, 12×FWHM, 6×FWHM, 4×FWHM. Spectra offset to aid clarity of peak separation.*

## S1.6. Closed-form overlap in an idealised two-peak model

Lorentzian tails imply that the variance (second moment) does not exist in the strict probabilistic sense, which is one reason “width” can be a poor summary statistic when peak tail behaviour introduces finite-window errors.^S3^ There is non-linear dependence on $\gamma$ and on the window geometry $(\Delta,W)$, (Equation S8 and Figure S3) i.e. overlap is intrinsically processing-sensitive under finite windows when linewidth changes.

### Setup: two peaks, one finite window

Let resonance A be centred at $\nu_{A}=0$, and resonance B at $\nu_{B}=\Delta>0$. Define a symmetric integration window around B:

|  | $\mathcal{R}_{B}=[\Delta-W,\Delta+W],$ | Equation S6 |
| --- | --- | --- |

with fixed $W>0$ (chosen a priori, e.g. as a multiple of the pre-apodisation linewidth).^S8^

***Figure S2****: Idealised two-peak geometry showing two Lorentzian resonances and the finite integration window $\mathcal{R}_{B}$.*

### Closed-form tail overlap (the arctan proof)

Use a unit-area Lorentzian for resonance A:

|  | $L(\nu;\nu_{0},\gamma)=\frac{1}{\pi}\frac{\gamma}{(\nu-\nu_{0})^{2}+\gamma^{2}}, \int_{-\infty}^{\infty} L(\nu;\nu_{0},\gamma)d\nu=1,$ | Equation S7 |
| --- | --- | --- |

where $\gamma$ is the HWHM (so $FWHM=2\gamma$).^S5,S6^

The fraction of area A that falls inside integration region B is:

|  | $F_{A\to B}(\gamma;\Delta,W)=\int_{\Delta-W}^{\Delta+W} L(\nu;0,\gamma) d\nu=\frac{1}{\pi}\left[ \arctan\left( \frac{\Delta+W}{\gamma} \right)-arctan\left( \frac{\Delta-W}{\gamma} \right) \right].$ | Equation S8 |
| --- | --- | --- |

***Figure S3****: Closed-form Lorentzian tail overlap $F_{A\to B}$ (fraction of resonance A falling within the finite integration window centred on resonance B) plotted versus exponential broadening strength $lb/R_{2}$. The matched-filter condition $lb=R_{2}$ corresponds to $lb/R_{2}=1$ (vertical dashed line), and the secondary axis indicates the associated linewidth scaling* $\gamma_{E}/\gamma_{0}=1+lb/R_{2}$

### Why exponential apodisation creates “integral tuning”

Under exponential apodisation, the tail fraction $F_{A\to B}(\gamma;\Delta,W)$ becomes $lb$-dependent, and the measured integral in $\mathcal{R}_{B}$ can shift with $lb$ even if the underlying chemical composition is unchanged. Baseline correction may change the apparent magnitude in practice (Section S1.4 and Section S3.4).

For Voigt profiles (post-Gaussian apodisation), exact closed-form overlap integrals are not available, but the reduced tail amplitude relative to pure Lorentzian is well-established.^S1,S9^ For the regime B ≈ R₂, the Voigt tail at large offsets |ν| ≫$\gamma_{G}$ retains asymptotic Lorentzian character (decaying as 1/ν²), but has significantly less $gb$-dependence.

**Figure S4**: Natural Lorentzian, exponential-broadened Lorentzian, and Gaussian-apodised Voigt under the same finite integration window (width in units of the natural FWHM), showing wing redistribution. See Table S1 and S2 for integral redistribution.

## S1.7. Numerical audit of the mapping *gb = cB^2^*

Other equivalence criteria (match Equivalent Noise Bandwidth (ENBW), match FWHM, match peak height, match SNR) do not necessarily result from the same functions.^S5,S7,S10^ ENBW is a standard signal processing metric used to quantify how much a window function alters the white noise in a spectrum. Specifically, the ENBW of a window function is the width of an ideal rectangular filter that would let through the same amount of white noise power as the window being used.

The goal is to demonstrate that the chosen constant $gb$ is not operationally sensitive to (i) the linewidth measurement definition and (ii) numerical details present in real pipelines (finite acquisition, discrete sampling, and zero-filling).

### What is being “matched” (and what is not)

We compare two processing choices applied to the same idealised one-sided exponential FID $s(t)=Ae^{-R_{2}t}e^{-i\omega_{0}t}$ for $t\geq0$.

1. Exponential apodisation: $w_{E}(t)=exp(-lb t)$. Under this idealised model it yields an effective decay rate $R_{2}+lb$, and provides the matched-filter benchmark for SNR in white Gaussian noise.
2. Pure Gaussian apodisation: $w_{G}(t)=exp(-gb t^{2})$ with $gb=c lb^{2}$. Here $c$ is chosen so that the Gaussian-apodised spectrum numerically matches the exponential-apodised spectrum under a specified linewidth metric.

The audit checks whether the definition-based choice $c=ln2$ is close to the “best” $c$ obtained under a pragmatic FFT-derived FWHM-matching rule, within the controlled one-peak model used here.

### Relevance of a discrete FFT audit for qNMR

Even when the underlying continuous-time mathematics is clean, NMR integrals are extracted from discrete spectra produced by a specific pipeline (finite acquisition length $T_{\text{acq}}$, sampling interval $dt$, optional zero-filling, and a chosen spectrum representation such as absorptive real vs magnitude).^S11^

The practical concern here is finite-window integration in crowded spectra. Therefore, it is useful to know whether the mapping between exponential and Gaussian “strength” changes materially once these digitisation steps are included.

### Summary of the numerical procedure

A synthetic one-peak FID is generated on a uniform grid $t_{n}=n dt$ for $n=0,\ldots,N-1$ with $N\approx T_{\text{acq}}/dt$, multiplied by a window, and Fourier transformed using an FFT (with optional zero-filling by an integer factor $zf$).

A linewidth proxy is extracted as the full-width at half-maximum (FWHM) using one of two estimators:

- linear: Linear interpolation at the half-maximum crossing.
- quadlog: Local quadratic fit to $log(\text{intensity})$ near the half-maximum crossing.

For fixed $(R_{2},lb,dt,T_{\text{acq}},zf)$ and a chosen spectrum representation, $c$ is optimised (bounded scalar search) to minimise $|\text{FWHM}_{E}-\text{FWHM}_{G}|$.

### Internal pipeline self-test

The script includes an internal self-test that compares the numerically measured FWHM for a pure exponential decay to a closed-form FWHM expected under the selected spectrum representation and then checks refinement consistency under a finer $(dt,T_{\text{acq}})$ grid. This reduces the risk that the inferred $c$ is an artifact of an inconsistent FFT convention or a broken FWHM measurement routine.


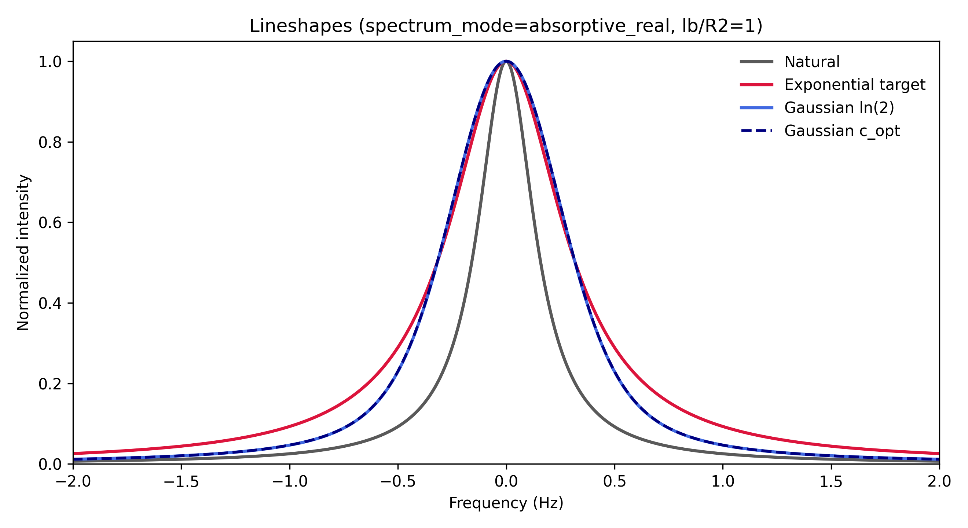


***Figure S5.*** *Absorptive-real spectra for a single peak under: natural exponential (no extra window), exponential apodisation (target), Gaussian apodisation with $c=ln2$, and Gaussian apodization with $c=c_{\text{opt}}$.*

## S1.8. Stage A: sampling and numerical convergence (varying dt)

Stage A tests how strongly the optimised constant $c_{\text{opt}}$ depends on sampling interval $dt$ when the frequency-bin spacing $\Delta f$ is fixed at $\Delta f=1/(T_{\text{acq}} zf)$ and $T_{\text{acq}}$ is set to a fixed long value. This design isolates time-domain discretisation effects from pure frequency-grid coarseness effects.

### What Stage A shows

Across the tested $dt$ values, $c_{\text{opt}}$ varies only modestly, and the difference between the “linear” and “quadlog” FWHM estimators is negligible (Figure S6, left). Over the same sweep, the FWHM mismatch incurred by using $c=ln2$ decreases with finer sampling.


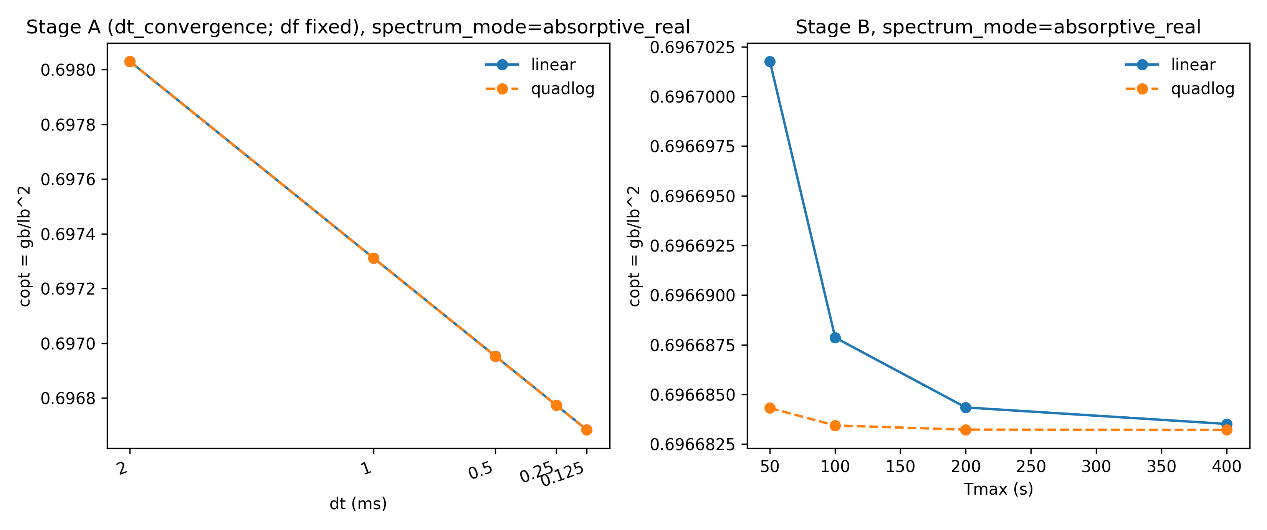


***Figure S6*** *Stage A (left): $c_{\text{opt}}$ vs $dt$. Stage B (right): $c_{\text{opt}}$ vs $T_{\text{acq}}$. Both estimators (“linear”, “quadlog”) are shown.*

### ln(2) residual (Stages A and B)

To quantify the practical impact of using Equation 4 as written, the script reports the FWHM mismatch (%) when forcing $c=ln2$ instead of using $c_{\text{opt}}$. The mismatch decreases with improved numerical conditions (finer sampling and longer acquisitions) and remains small across the tested range.


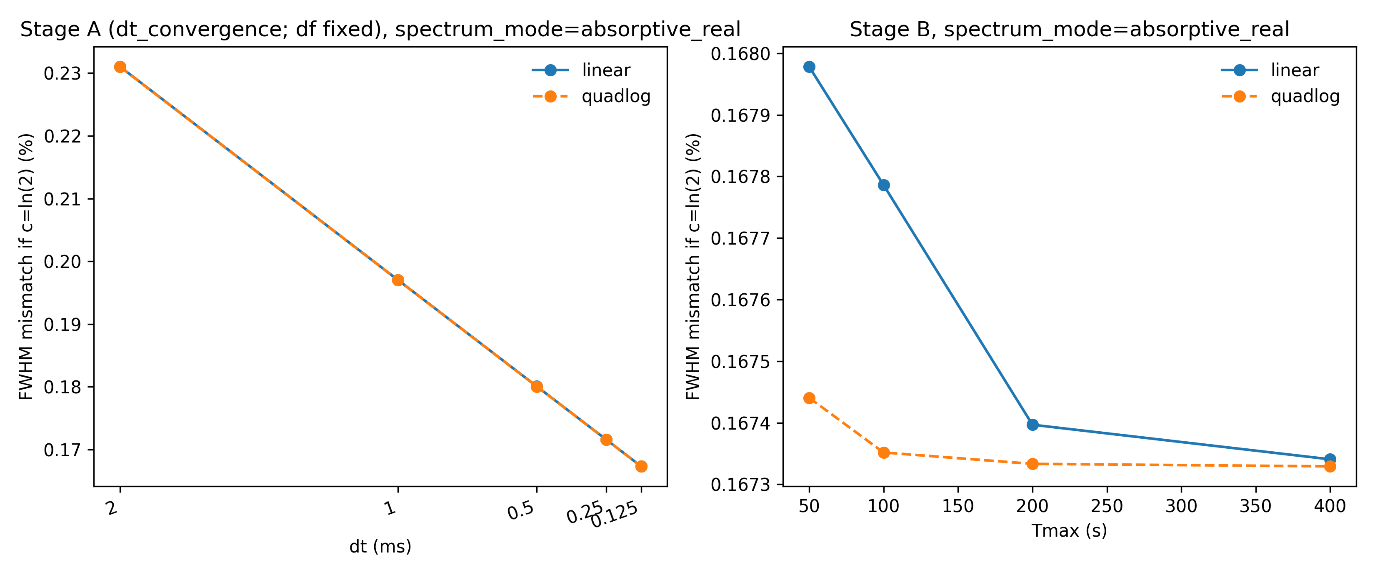
***Figure S7****. FWHM mismatch (%) when using $c=ln2$: Stage A (left) vs $dt$, Stage B (right) vs $T_{\text{acq}}$.*

## S1.9. Stage B: truncation and record length effects (varying T_acq_)

Stage B tests sensitivity to acquisition length $T_{\text{acq}}$ at fixed $dt$, i.e., it probes truncation and convergence with respect to how much of the windowed FID tail is retained. If the FID ends while the effective window is still appreciably nonzero, implicit multiplication by a hard step at $T_{\text{acq}}$ can introduce sinc-like artifacts and bias linewidth proxies. The Stage B panels in Figures S6 and S7 provide an empirical check that $c_{\text{opt}}$ and the ln(2) residual stabilise as $T_{\text{acq}}$ increases.

## S1.10. Parameter-regime sweep

The central motivation in this work is crowded-spectrum integration robustness, not a single-peak linewidth identity. Nevertheless, readers may ask whether Equation 4 behaves sensibly across broadening regimes (e.g., $B\neq R_{2}$) and what the SNR implications are when choosing Gaussian vs exponential weighting.

### Sweep over $\boldsymbol{B}\mathbf{/}\boldsymbol{R}_{\boldsymbol{2}}$

A sweep over ratios $B/R_{2}$ evaluates whether the optimised $c$ changes substantially as broadening is made weaker/stronger relative to the intrinsic decay. This directly addresses whether Equation 4 conceals a regime where the mapping becomes qualitatively misleading.


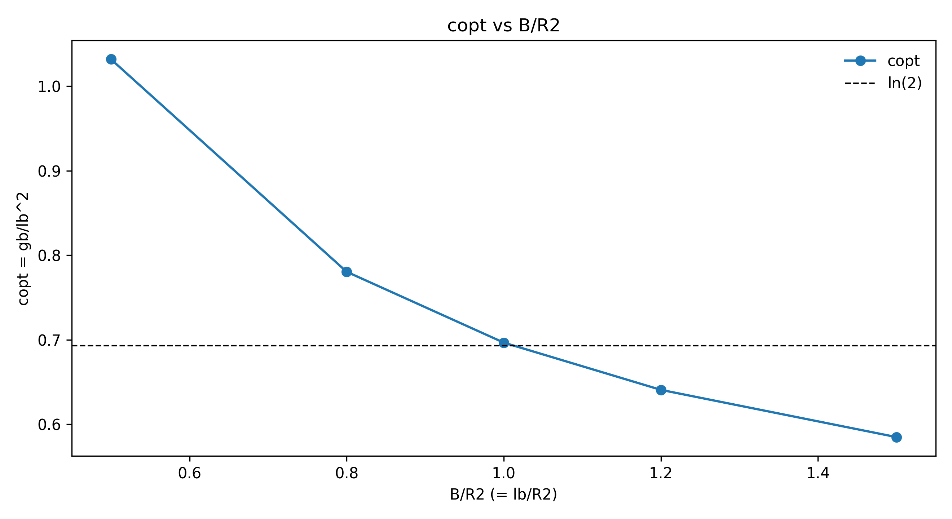
 ***Figure S8.*** $c_{\text{opt}}$ *vs $B/R_{2}$, with $c=ln2$ shown for reference.*


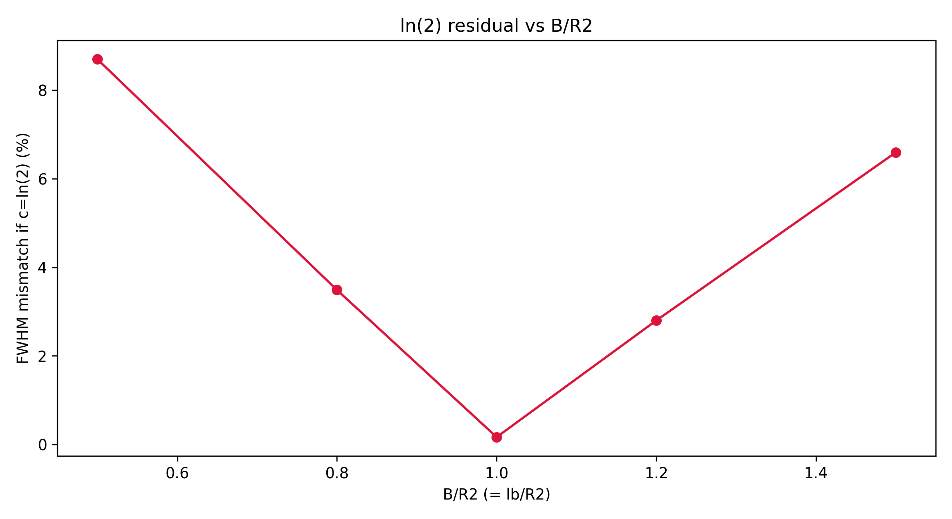


***Figure S9.*** *FWHM mismatch (%) when using $c=ln2$ instead of $c_{\text{opt}}$, plotted vs $B/R_{2}$.*

### Window-only noise metrics

Section S1.2 emphasised that exponential weighting is the matched filter for a purely exponential FID in white Gaussian noise, so Gaussian weighting is a deliberate mismatch chosen for robustness at a typically modest SNR penalty.^S12^

To make this trade-off concrete without tying the discussion to any one SNR estimator, which may vary with chosen software, the script computes window-only metrics derived from $\sum w$ and $\sum w^{2}$, comparing exponential vs Gaussian at $c=ln2$ and at $c=c_{\text{opt}}$.

- ENBW proxy (in bins): $N \frac{\sum w^{2}}{(\sum w)^{2}}$.
- RMS window factor: $Q_{\text{rms}}=\sqrt{\sum w^{2}/N}$.


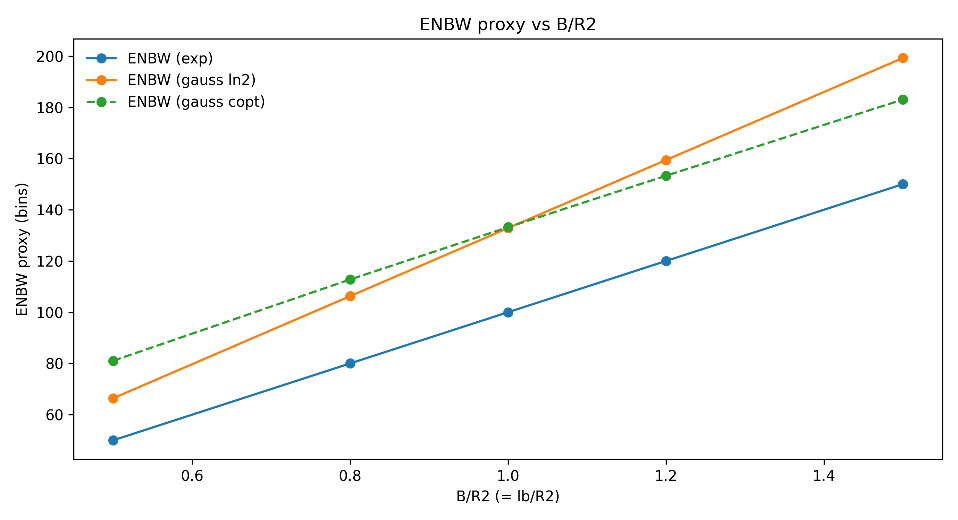

***Figure S10*** *ENBW proxy vs $B/R_{2}$ for exponential and Gaussian windows (using $c=ln2$ and $c=c_{\text{opt}}$).*


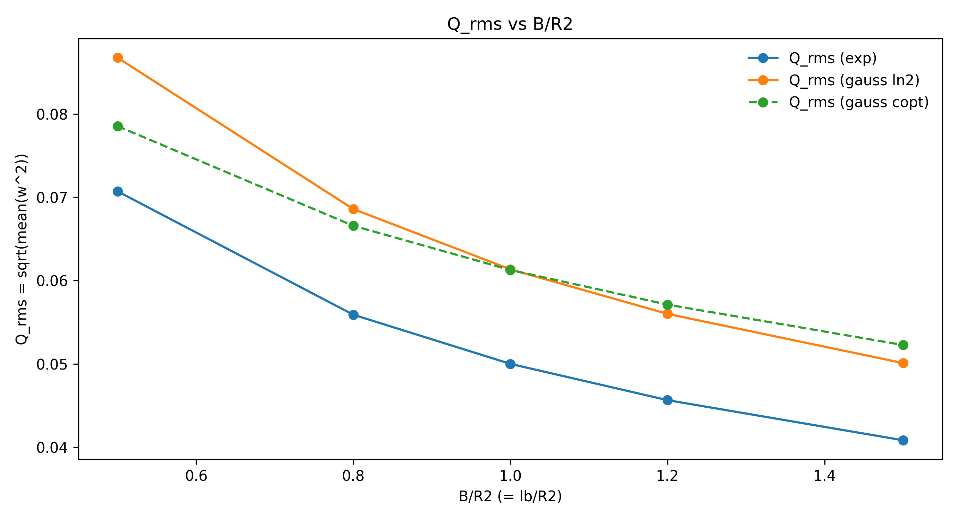


***Figure S11.*** $Q_{\text{rms}}$ *vs $B/R_{2}$ for exponential and Gaussian windows (using $c=ln2$ and $c=c_{\text{opt}}$).*


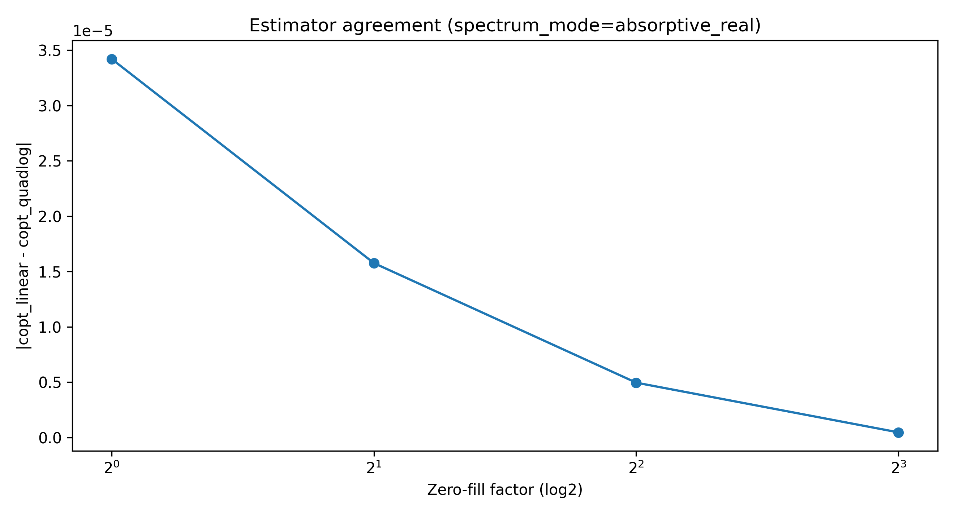

***Figure S12****: Estimator agreement vs zero-fill factor (log2): |$c_{\text{opt}}$_linear − $c_{\text{opt}}$_quadlog|, showing negligible dependence of inferred $c_{\text{opt}}$ on FWHM estimator choice across zero-filling (spectrum_mode = absorptivereal*).

## S1.12. Practical guidance

Equation 4 ($gb=B^{2}\ln2$) should be read as a standardised parameterisation of a pure Gaussian window, not as a claim that Gaussian and exponential apodisation are identical under all measures of “equivalent broadening”.

If a workflow demands a different equivalence criterion (e.g., FWHM matching under a specific FFT representation), a numerically optimised $c$ can be computed. The audit indicates that such a correction is typically small compared to the larger workflow choice being advocated: use pure Gaussian apodisation to reduce overlap sensitivity versus use exponential apodisation that directly increases Lorentzian wing prominence.

**Accordingly, we recommend reporting $\mathbf{B}$ (units s$\mathbf{}^{\mathbf{-1}}$) and the explicit window formula $\mathbf{w}_{\mathbf{G}}\mathbf{(t)=exp(-}\mathbf{B}^{\mathbf{2}}\mathbf{ln2}\mathbf{t}^{\mathbf{2}}\mathbf{)}$, and treating the numerical audit as a robustness check rather than a requirement for routine use.**

### Summary of key findings from the numerical audit

1. Equation 4 is stable across discretisation regimes: $c_{\text{opt}}$ remains close to $\ln2$ across the tested ranges of $dt$, $T_{\text{acq}}$, and $B/R_{2}$.
2. Estimator choice has minimal impact: “linear” and “quadlog” yield nearly identical $c_{\text{opt}}$ under the tested conditions.
3. Using $c=ln2$ produces small residual FWHM mismatch across the tested parameter space.
4. Gaussian apodisation shows a modest, predictable window-only noise penalty relative to exponential, consistent with the “conservative mismatch” framing.

### When to resort to this audit?

Most users should not need to run this audit at all: Equation 4 (main text) as written is sufficient for operational qNMR workflows.

The audit is valuable when: (a) Validating a new processing pipeline or software implementation; (b) Exploring extreme parameter regimes (e.g., very short $T_{\text{acq}}$ relative to $1/B$); (c) If there are questions regarding the choice $c=ln2$ vs other possible constants.

In all cases, the purpose of this audit is to demonstrate that Equation 4 is not arbitrary and users do not need to numerically optimise $c$ for every dataset.

## S1.13. Practical implementations note: software parameterisation differs

Equation 4 (main text) defines a specific time-domain window $w_{G}(t)=exp(-gbt^{2})$ beginning at $t=0$. Commercial NMR software sometimes parameterises “Gaussian” or “Lorentz–Gauss” processing using parameters that describe a *shifted* Gaussian maximum (fraction of acquisition time) and/or a combined exponential+Gaussian transform in addition to a pure $exp(-gbt^{2})$ decay from $t=0$.^S13–S15^ Other software only utilises a combined parameterisation, which are noted to be parameterised in different ways, and do not always allow for a pure Gaussian function to be defined.^S16,S17^ Users should not assume that a GUI parameter labelled “GB” equals the $gb$ in Equation 4.

**Translation/verification checklist.** (a) Confirm whether “Gaussian” found in the chosen software starts at $t=0$ with maximum amplitude and then decays (pure Gaussian apodisation), versus a shifted maximum used for resolution enhancement (Lorentz–Gauss);^S15-S17^ (b) Check the units expected by the software parameters (Hz vs dimensionless fractions of acquisition time), using vendor documentation or facility guides; (c) Run a controlled test: create a single exponential FID, apply the software window, and test whether the resulting frequency-domain line behaves as expected for the intended window family.

# S2. Peak Area Redistribution Table

***Table S1****. Reference Table for Peak Area Redistribution with exponential apodisation defined integral regions (× FWHM) and for broadening values (B). Integral region defined in terms of FWHM of non-apodised peak. Some values of interested are highlighted.*

| **Exponential** | | | | | | |
| --- | --- | --- | --- | --- | --- | --- |
| **Integral Region (xFWHM)** | **B = 0 R2** | **B = 0.5 R2** | **B = 1.0 R2** | **B = 2.0 R2** | **B = 3.0 R2** | **B = 4.0 R2** |
| **5** | 87.402 | 81.400 | 75.721 | 65.527 | 56.973 | 49.932 |
| **10** | **93.647** | 90.510 | **87.420** | 81.427 | 75.756 | 70.464 |
| **15** | 95.779 | 93.680 | 91.595 | 87.484 | 83.477 | 79.601 |
| **20** | **96.840** | 95.265 | **93.697** | 90.584 | 87.517 | 84.509 |
| **25** | 97.481 | 96.224 | 94.971 | 92.476 | 90.005 | 87.566 |
| **30** | 97.914 | 96.873 | 95.833 | 93.761 | 91.703 | 89.663 |
| **35** | 98.222 | 97.334 | 96.448 | 94.679 | 92.919 | 91.170 |
| **40** | **98.455** | 97.683 | **96.912** | 95.374 | 93.841 | 92.316 |
| **45** | 98.639 | 97.959 | 97.280 | 95.923 | 94.571 | 93.224 |
| **50** | 98.786 | 98.180 | 97.574 | 96.363 | 95.156 | 93.952 |
| **55** | 98.909 | 98.364 | 97.819 | 96.730 | 95.644 | 94.560 |
| **60** | 99.011 | 98.517 | 98.023 | 97.036 | 96.051 | 95.069 |

***Table S2****. Reference Table for Peak Area Redistribution with pure Gaussian apodisation at defined integral regions (× FWHM) and for broadening values (B). Integral region defined in terms of FWHM of non-apodised peak. Some values of interested are highlighted.*

| **Pure Gaussian** | | | | | | |
| --- | --- | --- | --- | --- | --- | --- |
| **Integral Region (xFWHM)** | **B = 0 R2** | **B = 0.5 R2** | **B = 1.0 R2** | **B = 2.0 R2** | **B = 3.0 R2** | **B = 4.0 R2** |
| **5** | 87.402 | 87.231 | 86.613 | 81.759 | 71.988 | 62.023 |
| **10** | **93.647** | 93.625 | **93.556** | 93.215 | 92.032 | 88.783 |
| **15** | 95.779 | 95.772 | 95.752 | 95.666 | 95.487 | 95.021 |
| **20** | **96.840** | 96.838 | **96.829** | 96.794 | 96.730 | 96.619 |
| **25** | 97.481 | 97.480 | 97.476 | 97.458 | 97.427 | 97.379 |
| **30** | 97.914 | 97.913 | 97.911 | 97.901 | 97.883 | 97.857 |
| **35** | 98.222 | 98.222 | 98.220 | 98.214 | 98.203 | 98.187 |
| **40** | **98.455** | 98.455 | **98.454** | 98.450 | 98.442 | 98.432 |
| **45** | 98.639 | 98.639 | 98.638 | 98.635 | 98.630 | 98.623 |
| **50** | 98.786 | 98.786 | 98.786 | 98.784 | 98.780 | 98.775 |
| **55** | 98.909 | 98.909 | 98.908 | 98.907 | 98.904 | 98.900 |
| **60** | 99.011 | 99.011 | 99.011 | 99.010 | 99.008 | 99.005 |

# S3. Case Study #1: Apodisation in Crowded Spectra

## S3.1 Stock Solutions

Reagents were purchased from commercial sources (Sigma Aldrich or Fluorochem) at the highest available grade, and were used without purification.

The following stock solutions were prepared:

Solution A: Si(TMS)_4_ (2.6 mg) made to 5 mL with CD_2_Cl_2_.
Solution B: Anethole (9.3 mg) made to 2 mL with Solution A.
Solution C: *m*-chloroperoxybenzoic acid (22.1 mg, 71.2 %) made to 2 mL with Solution A. *m*-chloroperoxybenzoic acid contains *m*-chlorobenzoic acid (28.8 %) for stability.

Reaction Initiation: Solution C (0.25 mL) was added to a mixture of Solution A (0.25 mL) and Solution B (0.1 mL) in an NMR tube and vigorously shaken.

*Reaction Concentrations:* Si(TMS)_4_ (1.6 mM), anethole (5.2 mM), *m*-chloroperoxybenzoic acid (19.0 mM) and *m*-chlorobenzoic acid (3.6 mM)

## S3.2 Spectrometer Details

NMR spectra for reaction monitoring and related experiments were acquired on a 400 MHz Bruker Ascend AVANCE III+ spectrometer equipped with a 5 mm BBO(H&F) nitrogen cryoprobe.

## S3.3 Reaction Monitoring

^1^H T_1_ measurements were estimated using FLIPS at 300 K prior to the reaction and T_1_^max^ was identified as 7.3 seconds. Parameters for NMR monitoring: NS=1, θ = 90°, τ_R_ = 40.0 s (5×T_1_^max^). Monitoring was commenced 38 s after addition of Stock Solution C.

## S3.4 NMR Processing Parameters

All FID and spectral processing were performed in MNova 14.0.0. Zero-filling was applied to double the size of the FID (32K to 64K). Zero and first order phase correction values were determined manually and remained consistent throughout. The spectra were manually phased and apodisation was applied as appropriate for the analysis. In MNova 14.0.0, the relationship between *lb* and *gb* is not as described in Equation 4 and 5 (main text, see Section S1.12 for discussion). However, the software provides an estimation of ‘integral-to-noise’ effect as a result of apodisation, which was used to identify a value of *gb* comparable to *lb*. Here, *lb* = 0.3 Hz and *gb* = 0.6 Hz where used. No baseline correction was applied to the spectrum to isolate the effects of apodisation. Integration of all spectra were carried out between the ranges in Table S3.

***Table S3****. Integration parameters used for raw data and averaged data.*

| ^1^H Resonance | Integral Minimum (ppm) | Integral Maximum (ppm) |
| --- | --- | --- |
| **2_a_** | 7.889 | 7.965 |
| **2_b_** | 7.670 | 7.729 |

## S3.5 Data Analysis

Baseline correction (polynomial order 5) across the whole spectrum introduces small additional deviations that are amplified when comparing spectra prior to and following apodisation.

This discrepancy can be attributed to the altered peak shape following apodisation, especially in the peak wings and surrounding baseline, and even minimal baseline correction algorithms. The extent of peak wing broadening is dependent on the intensity of each peak, and therefore its concentration and the point in the reaction. Additionally, other experimental factors, such as shimming, may introduce non-Lorentzian character to the peak shape, and will also be affected by further line shape changes caused by apodisation.

All of these factors will influence the optimum correction found by a baseline correction algorithm. The pure Gaussian function does not change the shape peak wings to the same extent as the exponential apodisation. It is anticipated that the baseline correction will act similarly on the spectra with no apodisation and Gaussian apodisation, with differences arising predominantly due to the increased SNR. When exponential apodisation is applied, it is anticipated that baseline correction will also interact with the extended peak wings, causing the % deviation to appear minimal at t = 1000 s. As described in the main text, this is likely the crosstalk between **2­_a_** and **4** to be equally matched. Deconvoluting these effects is outside the scope of this paper.

The trends outlined in Case Study 1, and the conclusions therein remain the same.


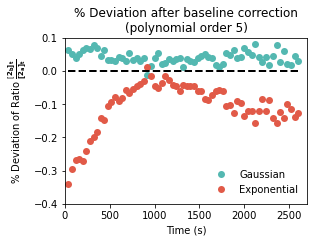


**Figure S12.** % Deviation of Integral ratio [**2_b_**]/[**2_a_**] over reaction time following baseline correction (polynomial order 5). Deviation in integral ratio following exponential apodisation follows similar shape to the chemical shift change of the neighbouring peak corresponding to acid **4** (see Figure 5, main text).]

**Figure S13.** Concentration**-**time profile of **2** and **4** over the reaction monitoring experiment.

# S4. Case Study #2: Maintaining Precision in Decongested Spectra

The example was carried out in conjunction with work by Flook and Lloyd-Jones.^S18^ For completeness, the experimental method is reproduced below. The data were reprocessed in the context of this work and details in Section S4.3 and S4.4 are specific to the analysis herein.

## S4.1 Stock Solutions

The following stock solutions were prepared:
Stock Solution A: HCl (1 mL, 37% w/w) was made to 2mL with H_2_O.
Stock Solution B: 4-bromobenzotrifluoride (22 mg, 0.1 mmol, internal standard) made to 10 mL with tetrahydrofuran.

Reaction: A mixture of both carbon isotopologues of 4-fluorophenyl MIDA boronate (~ 6mg, ~ 0.024 mmol) were added directly to an NMR tube with Stock Solution B (0.5 mL).
Reaction was initiated by addition of Stock Solution A (0.1 mL), followed by vigorous shaking.

*Reactant Concentrations*: 4-bromobenzotrifluoride (8 mM), 13C-MIDA boronate (29 mM), 12C-MIDA boronate (28 mM), HCl (0.1 M).

## S4.2 Spectrometer Details

NMR spectra for reaction monitoring and related experiments were acquired on a 400 MHz Bruker Ascend AVANCE III+ spectrometer equipped with a 5 mm BBO(H&F) nitrogen cryoprobe. The probe precluded the use of ^1^H decoupling sequences.

## S4.3 Reaction Monitoring

^19^F T_1_ measurements were estimated using FLIPS at 323 K prior to the reaction and T_1_^max^ was identified as 3.0 seconds. Parameters for NMR monitoring: NS=1, θ = 90°, τ_R_ = 24.5 s, with 8×T_1_^max^. Prior to initiation, NMR tube containing both isotopologues and Stock Solution B (0.5 mL) was heated to 323 K for 10 minutes. Monitoring was commenced 35 s after addition of Stock Solution A (0.1 mL).

## S4.4 NMR Processing Parameters

In post-acquisition processing, a contracting average was applied ($N_{\left( 4 \right)}^{con}$ = 150) in TopSpin 4.3.0.^S18^ All subsequent FID and spectral processing were performed in MNova 14.0.0. The spectra were zero-filled from 131K to 256K. Zero and first order phase correction values were determined manually and remained consistent throughout. Apodisation was applied when required. In MNova 14.0.0, the relationship between *lb* and *gb* is not as described in Equation 4 and 5 (main text, see Section S1.12 for discussion). However, the software provides an estimation of ‘integral-to-noise’ effect as a result of apodisation, which was used to identify a value of *gb* comparable to *lb*. Here, lb = 0.40 Hz and gb = 0.85 Hz which resulted in a SNR increase of 2.94 and 2.97 respectively.

A polynomial baseline correction of order 5 was then applied between -111.0 ppm and -115.4 ppm (substrates) and -62.0 ppm and -64.5 ppm (internal standard). Integration of all spectra were carried out between the ranges in Table S1.

***Table S4****. Integration parameters used for raw data and averaged data.*

| 19F Resonance | Integral Minimum (ppm) | Integral Maximum (ppm) |
| --- | --- | --- |
| Internal Standard | -63.741 | -62.816 |
| [^13^C_2_]-**5** | -114.055 | -113.747 |
| [^2^H_4_]-**5** | -114.644 | -114.313 |

## S4.5 Data Analysis

The isotopologue ratio (R) using the concentration data for [^13^C_2_]-**5** (B) and [^2^H_4_]-**5** (A) were plotted against fractional conversion (F), according to Equations S8 and S9. Calculations pertaining to the presented data are included in the associated data repository.

|  | $R=\frac{\left[ B \right]}{\left[ A \right]}$ | Equation S8 |
| --- | --- | --- |
|  | $F=1-\frac{\left[ A \right]+\left[ B \right]}{\left[ A \right]_{0}+\left[ B \right]_{0}}$ | Equation S9 |

# S5. Case Study #3: Apodisation Effects in Congested Spectra

Spectrum of Ergocalciferol was available from Reference 49 (main text) doi.org/10.1021/jm500734a (Supporting Information: jm500734a_si_002.zip, Example3_EC, Spectrum 1).

Unprocessed FID was zero-filled (256k) and Fourier transformed. Natural FWHM was estimated at 1.86 Hz using lineshape fitting provided by MNova with a Generalised Lorentzian lineshape.

Exponential apodisation of 0.6 Hz increased the FWHM to 2.46 Hz with a SNR increase of 3.8. Pure Gaussian apodisation of 1.25 Hz was required to obtain an equivalent SNR. The linewidth of the Gaussian-apodisation peak was estimated using a Lorentzian-Gaussian lineshape at 2.43 Hz.

**Figure S14.** Spectrum of Ergocalciferol. Natural FWHM estimated as 1.86 Hz using singlet at 0.634 ppm (indicated with arrow).

# S6. Data Availability

- Figures 1 – 6 (main text) and Figure S1 are generated by Apodisation Figures.ipynb.
- Redistribution tables (Section S2) were calculated in conjunction with Figure 3.
- Primary NMR data relating to Case Study 1 is provided, along with integral measurements used to generate Figure 5 (Panel C).
- Primary NMR data following post-acquisition signal-averaging (4 scans) relating to Case Study 2 is provided, along with integral measurements and fit data used to generate Figure 6 (Panel B).
- Figures S2 – S5 were generated by a deterministic script (MRC_manuscript_2026_SI_figs_FINAL.py) with a built-in self-test (--self-test) that records a version-stamped PASS report and run metadata.
- Source data for the convergence and sweep figures (Figures S6 – S12) are provided as CSV files generated by the audit script (out_gb_ln2_audit/tables/table01–table05).

# S7. References

S1 J. J. Olivero and R. L. Longbothum, *J. Quant. Spectrosc. Radiat. Transf.*, 1977, **17**, 233–236.

S2 Y. Wang, B. Zhou, R. Zhao, B. Wang, Q. Liu and M. Dai, *Mathematics*, 2022, **10**, 210.

S3 This work

S4 R. G. Spencer, *Concepts Magn. Reson. Part A*, 2010, **36A**, 255–265.

S5 R. R. Ernst, G. Bodenhausen, A. Wokaun, R. R. Ernst, G. Bodenhausen and A. Wokaun, *Principles of Nuclear Magnetic Resonance in One and Two Dimensions*, Oxford University Press, Oxford, New York, 1990.

S6 T. D. W. Claridge, *High-Resolution NMR Techniques in Organic Chemistry*, Pergamon, 1st Edition., 1999.

S7 J. C. Hoch and A. Stern, *NMR Data Processing*, Wiley, 1st edn., 1996.

S8 R. Nadjari and J.-P. Grivet, *J. Magn. Reson. 1969*, 1991, **91**, 353–361.

S9 A. K. Hui, B. H. Armstrong and A. A. Wray, *J. Quant. Spectrosc. Radiat. Transf.*, 1978, **19**, 509–516.

S10 F. J. Harris, *Proc. IEEE*, 1978, **66**, 51–83.

S11 J. C. Lindon and A. G. Ferrige, *Prog. Nucl. Magn. Reson. Spectrosc.*, 1980, **14**, 27–66.

S12 C. Cobas, J. A. García-Pulido, P. Mora, G. Selva and S. Sykora, *Magn. Reson. Chem. MRC*, 2025, **63**, 90–97.

S13 P. Kiraly, Improving Resolution or Sensitivity with the Apodization Slider, https://www.jeoljason.com/2021/12/16/improving-resolution-or-sensitivity-with-the-apodization-slider/, (accessed 8 February 2026).

S14 L. Castañar, G. D. Poggetto, A. A. Colbourne, G. A. Morris and M. Nilsson, *Magn. Reson. Chem.*, 2018, **56**, 546–558.

S15 *MestReNova 16 Manual*, 2025 MESTRELAB RESEARCH, 2025th edn.

S16 Re: AMMRL: Window function parameters - Bruker vs Varian from Gareth Morris on 2017-04-07 (Email Archives for April, 2017), https://www.ammrl.org/archives/April-2017/0005.html, (accessed 3 February 2026).

S17 *Topspin Processing Commands and Parameters User Manual,* Bruker Corporation, Version 007

S18 A. Flook and G. C. Lloyd-Jones, *J. Org. Chem.*, 2024, **89**, 16586–16593.
